# Evaluation of the artificial intelligence chatbots in frequently asked questions about retinitis pigmentosa: a comparative analysis between ChatGPT-4 and Gemini-2.0

**DOI:** 10.1186/s40942-025-00772-4

**Published:** 2025-11-28

**Authors:** Özlem Biçer, Esra Şahlı

**Affiliations:** 1 Dr Hulusi Alataş Elmadağ State Hospital, Ankara, Turkey; 2 Ankara University Graduate School of Health Science, Vision, Artificial Vision and Rehabilitation of Low Vision, Master Program, Ankara, Turkey; 3 Department of Ophthalmology, Ankara University School of Medicine, Ankara, Turkey

**Keywords:** Artificial intelligence, ChatGPT-4, Gemini-2.0, Readability, Retinitis pigmentosa

## Abstract

**Background:**

To evaluate the accuracy and readability of answers to common retinitis pigmentosa (RP) questions from the popular generative artificial intelligence (AI) chatbots ChatGPT-4 and Gemini-2.0.

**Methods:**

In March 2025, frequently asked questions about RP was entered to Google search tool, and the websites appearing on the first search page were selected for enrollment in the study. ChatGPT-4 and Gemini-2.0 were then prompted to generate responses about RP in both standard and simplified formats. To generate the simplified response, the following request was added to the prompt: ‘Please provide a response suitable for the average American adult, at a sixth-grade comprehension level.’ The AI chatbots’ responses to 30 questions about RP, frequently asked by patients, were evaluated by two ophthalmologists using a five-point Likert scale, with scores ranging from 1–5. Additionally, 8 readability indices, including Average Reading Level Consensus Calculator (ARLC), Automated Readability Index (ARI), Flesch Reading Ease (FRE), Gunning Fog Index (GFOG), Flesch–Kincaid Grade Level (FKGL), Coleman–Liau Index (CL), Simple Measure of Gobbledygook (SMOG), and Forcast Readability Formula (FRF) were calculated using an online calculator, Readabilityformulas.com, to assess the ease of comprehension of each answer.

**Results:**

No significant difference showed in accuracy both standard and simplified AI chatbot responses (*p* = 0.557, *p* = 0.090). In particular, almost all readability indices suggest that standard AI chatbot responses require a higher level of education for comprehension, whereas simplified responses require a lower level of education. Although Gemini-2.0 standard responses were more readable than ChatGPT-4 standard responses according to ARI, GFOG and FRF scores (*p* = 0.014, *p* = 0.040, and *p* = 0.001), Gemini-2.0 simplified responses were more readable than ChatGPT-4 simplified responses solely according to FRF scores (*p* = 0.016).

**Conclusions:**

This study shows that ChatGPT-4 and Gemini-2.0 can provide patients with an avenue to access comprehensive and accurate information about, tailored RP to their educational level.

**Supplementary Information:**

The online version contains supplementary material available at 10.1186/s40942-025-00772-4.

## Background

Artificial Intelligence (AI) is a field of technology that prioritizes computer technology and seeks to reduce the need for human interaction. Currently, numerous fields of research, technology, and medicine have started to apply artificial intelligence [1, 2]. In the realm of medical sciences, artificial intelligence has various applications, including the selection of a physician based on patient symptoms, patient diagnosis, and medication development [3].

Another application of AI is the widespread use of AI chat programs like ChatGPT and Gemini, with which society interacts most today. Patients with RP about low vision concerns frequently have questions that require nuanced answers. To that end, patient education is one avenue that may see remarkable change, as patients equipped with AI technology can produce answers to their very specific questions. Rather than relying on difficult-to-navigate, static websites such as Wikipedia or WebMD, patients can now input seemingly any question and receive very focused responses [4, 5]. This may serve as an excellent resource for patients learning more about their health and options. Given this broad access to AI technology, it therefore becomes important to evaluate the quality of these responses.

To the best of our knowledge, there is no study in the literature that has investigated the effectiveness of chatbots for RP. In this study, we aimed to evaluate and compare the performances of two popular chatbots (ChatGPT-4, and Gemini-2.0) in responding to queries to RP-related questions. We thoroughly assessed the accuracy and readability of each AI chatbot’s responses.

## Methods

This original research study was planned as an observational, cross-sectional study. Ethical approval was not required as no human or animal participants were involved.

In the literature, it is reported that 90% of search-engine users only viewed the first three pages of search results [6]. Therefore, these websites were first analysed. Links to each webpage and the title of each content are shown in Table 1. A search was conducted using the Google Search Tool to identify websites that responded to the search term “frequently asked questions about RP”. The 30 questions were selected by the authors, based on previous studies on the accuracy and readability of chatbots, without a specific rationale for choosing 30 questions [7, 8]. Similar or duplicate questions were excluded from the study. The questions were submitted once each to ChatGPT-4 (OpenAl, California), and Gemini-2.0 (Alphabet Inc., California, USA) on March 31, 2024, and the responses were recorded. For every new search request made in chatbots, a separate conversation page was initiated to prevent past queries from influencing the responses to subsequent queries. Since Large Language Models (LLMs) can generate several responses to a given question, only the initial response was documented for analysis. Additionally, the ability of LLMs to simplify texts for lower educational levels was evaluated. To assess this, the models were given their initial responses with an additional prompt to generate a new response: ‘Please provide a response suitable for the average American adult, at a sixth-grade comprehension level.’

**Table 1:** Supplemental table S1: Comprehensive list of web resources for frequently asked questions about retinitis pigmentosa

1	https://irisvision.com/frequently-asked-questions-about-retinitis-pigmentosa/?srsltid=AfmBOorKZPXFaCLZd51omXoqg0-rUp2CHkWgaYXjPdgtcQn29SPFBB2P
2	https://www.restorevisionclinic.com/vision-restoration-resources/retinitis-pigmetosa-faq
3	https://www.guidedogs.org.uk/getting-support/information-and-advice/eye-conditions/retinitis-pigmentosa/living-with-retinitis-pigmentosa/
4	https://www.medindia.net/health/conditions/retinitis-pigmentosa-faqs.htm

The readability of each response was assessed using https://readabilityformulas.com/free-readability-formula-tests.php, an online readability calculator [9]. This process involved analyzing the texts to count the number of characters, words, syllables, and sentences in order to assess the ease of comprehension and level of education needed to understand the text. Subsequently, 8 readability indices, including Average Reading Level Consensus Calculator (ARLC), Automated Readability Index (ARI), Flesch Reading Ease (FRE), Gunning Fog Index (GFOG), Flesch–Kincaid Grade Level (FKGL), Coleman–Liau Index (CL), Simple Measure of Gobbledygook (SMOG), and Forcast Readability Formula (FRF) Index, were used for this purpose.

The FRE score ranges from 1 to 100, with a higher value directly correlated with how easy the text is to read. The FKGL, GFOG, SMOG Index, and CL Index are tools that approximate the U.S. grade level needed for understanding a text [10]. Table 2 summarizes formulas used for each index. ARLC processed the text through eight popular readability formulas (Linsear Write Formula, SMOG Index, CL Index, FKGL, GFOG, FRES, ARI, FRF) and averaged out the results to yield an approximate reading difficulty score.

**Table 2:** Readability formulas

Readability Index	Formula
ARI	(4.71 * (characters/words)) + (0.5 * (words/sentences)) − 21.43
FRE	206.835 – (1.015 × total words/total sentence) – (84.6 × total syllables/total word)
GFOG	0.4 * ((words/sentences) + (percentage of complex words))
FKGL	(0.39 × total words/total sentence) + (11.8 × total syllables/total word) – 15.59
CL	0.0588 × (characters/100 words) – 0.296 × (sentences/100 words) – 15.8
SMOG	3 + (square root of ≥ 3-syllable words count/30 sentences)
FRF	20 - (# of Single Syllable Words x 150 / # of Words x 10)
ARLC	Average of results according to 8 formulas

The AI-generated responses were evaluated separately by two experienced ophthalmologists (OB, ES) using the five-point Likert scale (1, Strongly disagreed; 2, Disagreed; 3, Not in agreement or disagreement; 4, Agreed; 5, Strongly agreed). The two experts independently provided the evaluations. The ophthalmologists decided that the consensus score as the final agreed-upon rating after discussion and using the criteria to settle differences in the Likert scale evaluations.

Statistical analysis was performed using Python 3.11.1 (Python Software Cooperation, Wilmington, DE, USA), utilizing libraries including pandas, SciPy, statsmodels, and matplotlib. Descriptive statistics are expressed as the mean ± standard deviation or median (minimum-maximum) for accuracy scores and readability scores. The Shapiro-Wilk test, kurtosis, and skewness values were used to assess normality. A normal distribution was accepted if kurtosis and skewness values were between (-1.5) and (+ 1.5). Levene’s test was used to examine variance homogeneity. Student t-test and Mann Whitney U test were applied to determine the interactions between chatbots for accuracy scores and readability scores. The results were evaluated at a significance level of *p* < 0.05.

## Results

All questions along with their corresponding responses generated by ChatGPT-4 and Gemini-2.0 are provided in detail in Supplemental Digital Content 1. (See appendix, Supplemental Digital Content 1, which displays the questions and answers generated)

Mean (± standard deviation) and median (minimum-maximum) accuracy scores, readability scores for ChatGPT-4 and Gemini-2.0 were determined, with direct comparison between groups (Tables 3 and 4).

**Table 3:** Summary of likert scores and readability indices of standard ChatGPT-4 and Gemini-1.5 responses

	ChatGPT-4	Gemini-1.5	*P* value
	mean-SD median (min-max)	mean-SD median (min-max)	
Accuracy			
Likert scores (1–5)	4.97 ± 0.18 5 (4–5)	4.87 ± 0.57 5 (2–5)	*p* = 0.557*
Readability indices			
ARLC	12.01 ± 1.73 12.08 (7.71–15.88)	11.25 ± 1.20 11.36 (8.96–14.28)	*p* = 0.053^+^
ARI	11.39 ± 2.38 11.46 (4.91–17.58)	10.3 ± 1.62 10.56 (7.63 ± 15.36)	*p* = 0.014*
FRES	36.17 ± 13.37 38.0 (10.0–69.0)	41.33 ± 10.28 44.5 (16.0–60.0)	*p* = 0.099^+^
GFOG	13.65 ± 2.42 13.2 (9.3–20.0)	12.36 ± 1.69 12.35 (9.60–15.8)	*p* = 0.040*
FKGL	11.08 ± 2.23 11.0 (5.96–17.02)	10.32 ± 1.46 10.17 (7.58–14.22)	*p* = 0.127^+^
CL	13.84 ± 2.43 13.98 (6.84–18.15)	12.99 ± 1.89 12.82 (9.56–18.66)	*p* = 0.136^+^
SMOG	9.93 ± 1.73 9.77 (6.14–15.93)	9.45 ± 0.98 9.75 (7.51–11.40)	*p* = 0.308*
FRF	12.76 ± 0.89 12.80 (10.50–14.86)	11.94 ± 0.82 11.82 (10.33–13.98)	*p* = 0.001^+^

ARLC: Average Reading Level Consensus Calculator, ARI: Automated Readability Index, FRES: Flesch reading ease score, GFOG: Gunning Fog, FKGL: Flesch-Kincaid grade level, CL: Coleman-Liau, SMOG: Simple Measure of Gobbledygook, and FRF: Forcast Readability Formula

*Mann Whitney U Testi, ^+^ Student T Testi

**Table 4:** Summary of likert scores and readability indices of simplified ChatGPT-4 and Gemini-1.5 responses

	ChatGPT-4	Gemini-1.5	*P* value
	mean-SD median (min-max)	mean-SD median (min-max)	
Accuracy			
Likert scores (1–5)	4.97 ± 0.18 5 (4–5)	4.83 ± 0.38 5 (4–5)	*p* = 0.090*
Readability indices			
ARLC	6.73 ± 1.3 6.58 (4.54–9.54)	6.72 ± 0.97 7.17 (5.11–8.12)	*p* = 0.953*
ARI	6.48 ± 1.6 6.78 (3.35–9.98)	6.6 ± 1.35 6.48 (4.4 ± 9.5)	*p* = 0.743^+^
FRES	75.97 ± 6.89 76.0 (60.0–89.0)	76.83 ± 4.53 76.0 (71.0–88.0)	*p* = 0.584*
GFOG	6.94 ± 1.54 6.6 (4.9–11.6)	6.96 ± 1.27 6.95 (4.2–9.7)	*p* = 0.478*
FKGL	5.94 ± 1.54 6.02 (3.15–9.08)	5.95 ± 1.19 6.17 (4.16–7.81)	*p* = 0.988^+^
CL	7.51 ± 1.11 7.56 (4.79–10.37)	7.27 ± 0.78 7.22 (5.45–8.71)	*p* = 0.329^+^
SMOG	5.89 ± 1.23 5.7 (4.32–9.0)	6.22 ± 1.01 6 (4.33–7.6)	*p* = 0.147*
FRF	9.49 ± 0.56 9.57 (8.47–10.6)	9.18 ± 0.41 9.14 (8.0–9.86)	*p* = 0.016^+^

ARLC: Average Reading Level Consensus Calculator, ARI: Automated Readability Index, FRES: Flesch reading ease score, GFOG: Gunning Fog, FKGL: Flesch-Kincaid grade level, CL: Coleman-Liau, SMOG: Simple Measure of Gobbledygook, and FRF: Forcast Readability Formula

*Mann Whitney U Testi, ^+^ Student T Testi

In particular, almost all readability indices suggest that standard AI chatbot responses require a higher level of education for comprehension, whereas simplified responses require a lower level of education. Although Gemini-2.0 standard responses were more readable than ChatGPT-4 standard responses according to ARI, GFOG and FRF scores (*p* = 0.014, *p* = 0.040, and *p* = 0.001) (Table 3), Gemini-2.0 simplified responses were more readable than ChatGPT-4 simplified responses solely according to FRF scores (*p* = 0.016) (Table 4).

## Discussion

Advances in natural language processing have enabled conversational robots to play a promising role in healthcare, with potential applications in disease prevention, diagnosis, treatment, monitoring and patient support. Numerous LLM Chatbots inception led to numerous speculations about which AI chatbot would deliver greater accuracy. Each AI model has distinct advantages and disadvantages making them appropriate for various applications and use cases [11]. To date, few studies have objectively compared the accuracy of responses, with even fewer studies focusing on the medical field [12]. To the best of our knowledge, this is the first comparative cross-sectional study evaluating the performance of ChatGPT-4 and Gemini-2.0 on RP-related questions. Our study revealed that the results highlight the ability of both AI chatbots to customize responses about RP to suit each patient’s comprehension level.

Among the currently most utilized chatbots, ChatGPT stands at the forefront of the most popular conversational assistants. ChatGPT is an AI chatbot based on LLM trained with a large number of parameters until September 2021. ChatGPT version 3.5 model was released in November 2022 and then the newest developed model ChatGPT version 4 was released in March 2023. Bard rebranded as Gemini in February 2024 is trained on real-time data from web text and public dialogue, and it is powered by the LaMDA language model that has been used since March 2023 [13]. 

The simplified responses provided by both AI chatbots consistently showed better readability, requiring lower education levels for full comprehension compared to their standard versions. Among the standard-format outputs, Gemini-2.0 generally achieved lower scores on readability metrics such as ARI, GFOG, and FRF, suggesting that it may be slightly better at producing more accessible content than ChatGPT-4. However, when focusing solely on the simplified responses, Gemini-2.0 maintained a measurable advantage only in FRF scores. This pattern indicates that while Gemini-2.0 may demonstrate modest improvements in certain readability measures, the overall practical significance of these differences appears limited.

The responses from AI chatbots are critical in scenarios where misinformation could have serious consequences for patient care or medical decision-making. These findings underscore the importance of readability in patient education materials, highlighting that generative AI models like ChatGPT-4 and Gemini-2.0 can effectively tailor content to varying educational levels. This adaptability is crucial, given the diverse educational backgrounds of patients seeking information online. The study also emphasizes the utility of simplified communication in healthcare contexts, potentially enhancing patient understanding and engagement. This differentiation highlights the importance of adapting chatbot applications to specific user needs, whether for detailed professional discourse or straightforward patient communication.

A study conducted by Lim et al. found a significant difference in accuracy between three LLMs in a study comparing LLMs’ performances for myopia care: ChatGPT-4, 80.6%; ChatGPT-3.5, 61.3%; and Google Bard, 54.8% [14]. In a 2024 study, Ichhpujani et al. investigated the appropriateness and readability of AI chatbots’ responses to for surgical treatment of glaucoma. It was found that ChatGPT-3.5 provided more accuracy and complexity than Google Bard [15]. 

In our study, it was showed that both AI models performed similarly in terms of accuracy, with no statistically significant differences found between their standard and simplified response formats. This suggests that both platforms are dependable sources for providing accurate information to patients looking for guidance about RP.

In terms of readability, the simplified responses produced by both AI chatbots offered a clear advantage, requiring a lower educational level for comprehension compared to their standard versions. This finding highlights the value of simplified outputs in making complex medical information more accessible to a broader audience.

The main limitation of our study was that it involved a small number of questions focused solely on the topic of RP. Deep learning-based chatbots continuously update their responses using newly available online data, leading to variability in answers over time. This dynamic nature complicates control and consistency. Notably, another limitation of this study is each question was asked only once, and only the initial responses were evaluated. Therefore, the aspect of repeatability of responses was not addressed in this research. It should be kept in mind that language chatbots may be updated frequently, therefore the responses they provide could vary.

In conclusion, both AI models exhibit comparable accuracy in their responses, with no statistically significant differences noted in either the standard or simplified answer formats. Future advancements should aim to combine the strengths of both models, creating hybrid models that optimize accuracy, accessibility, and adaptability in AI-assisted healthcare communication.

## Supplementary Information

Below is the link to the electronic supplementary material.

Supplementary Material 1

Supplementary Material 2

Supplementary Material 3

Supplementary Material 4

## Data Availability

No datasets were generated or analysed during the current study.
