# Supplementary material for: Evaluation of the artificial intelligence chatbots in frequently asked questions about retinitis pigmentosa: a comparative analysis between ChatGPT-4 and Gemini-2.0
Source: Int J Retina Vitreous. 2025 Nov 28;12:1. doi: 10.1186/s40942-025-00772-4 (PMC12763912; doi:10.1186/s40942-025-00772-4)
Supplement: Supplementary file 1 — Supplementary Material 1 [file 40942_2025_772_MOESM1_ESM.docx]

What is Retinitis Pigmentosa?

**Retinitis Pigmentosa (RP)** is a group of inherited eye diseases that cause **progressive degeneration of the retina**, the light-sensitive tissue at the back of the eye. It primarily affects the **photoreceptor cells**—**rods** and **cones**—which are responsible for vision in low light and color/detail, respectively.

**Key Features:**

- **Night blindness** (nyctalopia): Often one of the earliest symptoms due to rod cell degeneration.
- **Peripheral vision loss**: Commonly described as "tunnel vision" as peripheral vision gradually diminishes.
- **Central vision loss**: May occur later in the disease, particularly when cone cells are affected.
- **Photopsia**: Patients may report seeing flashes or flickering lights.
- **Fundus changes**: Classic findings include **bone-spicule pigmentation**, **attenuated retinal vessels**, and **waxy pallor of the optic disc**.

**Causes:**

- **Genetic mutations** in over 60 different genes.
- Inheritance patterns:
  - **Autosomal recessive** (most common)
  - **Autosomal dominant**
  - **X-linked recessive** (often more severe)

**Diagnosis:**

- **Electroretinography (ERG)**: Measures electrical activity of the retina, showing reduced rod and/or cone response.
- **Visual field testing**: Reveals peripheral vision loss.
- **OCT (Optical Coherence Tomography)**: Shows retinal thinning and photoreceptor loss.
- **Genetic testing**: Identifies specific mutations for diagnosis and counseling.

**Management:**

- No definitive cure yet, but:
  - **Low vision aids** and **rehabilitation** help with functional vision.
  - **Vitamin A** supplementation (in selected cases and under guidance).
  - **Gene therapy**: Approved for RPE65 mutation (e.g., *Luxturna*).
  - **Retinal implants** and **research into stem cells and neuroprotection** offer future hope.

If you'd like, I can tailor this summary for a clinical audience, presentation slide, or patient-friendly explanation—just let me know!

What causes you to get Retinitis Pigmentosa?

Retinitis Pigmentosa (RP) is caused by **genetic mutations** that affect the retina’s ability to respond to light. These mutations lead to the progressive degeneration of the photoreceptor cells—**primarily rods** (responsible for night and peripheral vision), and eventually **cones** (responsible for central and color vision). Here's a breakdown of the causes:

### 🔬 ****Genetic Causes****

RP is primarily **inherited**, and can follow different genetic patterns:

1. **Autosomal Recessive RP**
   - Both parents are carriers, but usually unaffected.
   - Often more severe, earlier onset.
   - Most common form globally.
2. **Autosomal Dominant RP**
   - One affected parent can pass the gene to children.
   - Tends to be milder and slower in progression.
3. **X-linked RP**
   - Caused by mutations on the X chromosome.
   - Mainly affects males; females may be carriers with mild symptoms.
4. **Sporadic (No family history)**
   - De novo mutations; no known affected family members.
   - Still genetically based but without clear inheritance.

### 🧬 Common Mutated Genes in RP

- **RHO** (rhodopsin) – common in autosomal dominant RP.
- **USH2A** – associated with Usher syndrome (RP with hearing loss).
- **RPGR** – common in X-linked RP.
- **CRB1, PDE6A/B, RPE65**, and many others.

Over 60 different genes are linked to RP—each mutation can impact different parts of the visual cycle or photoreceptor cell function.

### 🧏 Syndromic vs Non-Syndromic RP

- **Non-syndromic RP**: Only affects the eyes.
- **Syndromic RP**: Part of a broader syndrome (e.g., **Usher syndrome**, **Bardet-Biedl syndrome**) affecting hearing, metabolism, or other organs.

Let me know if you want a gene-specific breakdown or how RP relates to syndromes you’re studying.

Can Retinitis Pigmentosa be passed down through heredity?

Yes, **Retinitis Pigmentosa (RP)** can definitely be passed down through heredity. It’s a group of inherited disorders that affect the retina's ability to respond to light, leading to progressive vision loss.

There are **three main inheritance patterns** for RP:

1. **Autosomal dominant RP**
   - Affected person inherits one mutated gene from one parent.
   - Tends to have a **later onset** and **slower progression**.
   - Each child has a **50% chance** of inheriting the condition.
2. **Autosomal recessive RP**
   - A person inherits two mutated genes, one from each parent (who are usually unaffected carriers).
   - Tends to have an **earlier onset** and **more severe progression**.
   - **25% chance** of an affected child if both parents are carriers.
3. **X-linked RP**
   - Caused by a mutation on the X chromosome.
   - **Males** are usually affected; **females** are typically carriers but may have mild symptoms.
   - Sons of carrier mothers have a **50% chance** of being affected.

There are also **sporadic cases** with no known family history, often due to **new (de novo) mutations**.

Do you want details on genetic testing or counseling options for RP?

Can Retinitis Pigmentosa be caused by injury or accident?

No, Retinitis Pigmentosa (RP) is not caused by injuries or accidents. It is a genetic condition characterized by progressive degeneration of photoreceptors (rods and cones) in the retina, usually inherited through autosomal dominant, autosomal recessive, or X-linked genetic patterns.

While trauma or injuries to the eye can cause retinal damage or vision loss, this type of acquired retinal damage would not be classified as Retinitis Pigmentosa.

RP typically manifests with symptoms such as:

- Night blindness (nyctalopia)
- Gradual loss of peripheral vision (tunnel vision)
- Progressive visual field loss leading to eventual central vision impairment
- Characteristic pigmentary changes in the retina visible upon examination

Injury or trauma can cause retinal detachment, hemorrhages, scarring, or other types of retinal damage, but these would result in different clinical presentations and would not resemble the genetic condition of RP.

Thus, RP itself is purely genetic and cannot be triggered by external injury or trauma.

What are the symptoms of Retinitis Pigmentosa?

Retinitis pigmentosa (RP) is a group of inherited retinal disorders characterized by progressive degeneration of photoreceptors (rods and cones), typically leading to vision loss.

Common symptoms include:

### Early symptoms:

1. **Night blindness (nyctalopia)**:
   - Usually the earliest and most noticeable symptom.
   - Difficulty adapting to low-light conditions.
2. **Peripheral vision loss (tunnel vision)**:
   - Gradual constriction of the visual field, often progressing inward.

### Later symptoms:

1. **Progressive visual field narrowing**:
   - The visual field constricts significantly, leading to severe peripheral vision loss.
2. **Difficulty seeing in dim or poorly lit environments**:
   - Significant challenges navigating in the evening or indoors under dim lighting.
3. **Photopsia (flashes of light)**:
   - Some patients experience intermittent flashes or sparks of light.
4. **Photophobia (sensitivity to bright light)**:
   - Increased discomfort or difficulty seeing in bright conditions.
5. **Loss of color vision**:
   - Difficulty distinguishing between certain colors, typically noticeable in advanced stages.
6. **Reduced visual acuity (central vision loss)**:
   - Initially central vision is preserved, but over time, deterioration may occur, making detailed tasks challenging.

### Associated ocular findings (seen by clinicians):

- **Bone-spicule pigmentation** on the retina (classical sign).
- **Pale optic disc** due to optic nerve atrophy.
- **Retinal vessel attenuation** (thinning and narrowing of retinal blood vessels).
- **Cystoid macular edema** in some patients, potentially affecting central vision.
- **Posterior subcapsular cataracts**.

Symptoms typically progress slowly and worsen with age, but the rate of progression varies significantly between individuals.

How can Retinitis Pigmentosa be diagnosed?

Retinitis Pigmentosa (RP) can be diagnosed using a combination of clinical evaluation, imaging, and specialized functional tests:

### 1. ****Clinical History and Symptoms****

- Night blindness (nyctalopia) is often the earliest symptom.
- Progressive peripheral vision loss ("tunnel vision").
- Family history of RP or inherited retinal dystrophies.

### 2. ****Fundoscopic Examination****

Characteristic clinical signs include:

- **Bone-spicule pigmentation**: Pigment deposits in the peripheral retina.
- **Retinal vessel attenuation**: Narrowing of retinal arteries.
- **Optic disc pallor**: Wax-like, pale appearance due to nerve degeneration.

### 3. ****Visual Field Testing****

- Typically reveals peripheral visual field constriction progressing towards central vision loss.
- Usually performed using automated perimetry (Humphrey visual field test) or Goldmann perimetry.

### 4. ****Electroretinography (ERG)****

- The **gold standard** diagnostic tool.
- Shows reduced or absent rod and cone responses (initially rods, later cones).
- Helps to determine severity and extent of photoreceptor dysfunction.

### 5. ****Optical Coherence Tomography (OCT)****

- Imaging shows thinning of photoreceptor layers, specifically the outer retinal layers, indicative of photoreceptor loss.
- Can assess central macular edema or macular atrophy.

### 6. ****Fundus Autofluorescence (FAF)****

- Reveals patterns of increased or decreased autofluorescence corresponding to retinal pigment epithelial dysfunction.
- Helps identify areas of disease progression.

### 7. ****Genetic Testing****

- Confirms the genetic cause, subtype, inheritance pattern, and assists in prognosis and genetic counseling.
- Important for identifying candidates for potential gene therapy or clinical trials.

### Differential Diagnoses to Consider:

- Usher syndrome (RP with hearing loss)
- Bardet-Biedl syndrome
- Leber congenital amaurosis
- Cone-rod dystrophy

An accurate diagnosis of RP typically requires integrating clinical findings, imaging, ERG results, and genetic analysis to confirm the specific type and guide patient management.

How does Retinitis Pigmentosa affect vision?

Retinitis Pigmentosa (RP) is a group of inherited retinal disorders that gradually lead to vision loss due to progressive degeneration of the photoreceptor cells in the retina, especially the rods (responsible for peripheral and night vision) and later the cones (responsible for central and color vision). Here’s how RP typically affects vision:

### 1. ****Night Blindness (Nyctalopia)****

- **Early symptom** due to degeneration of rod cells.
- Difficulty seeing in low-light or dark environments.

### 2. ****Loss of Peripheral Vision****

- Often described as **"tunnel vision."**
- Peripheral vision gradually narrows, making it difficult to see objects to the side.
- In advanced stages, this constricted field can reduce to a small central island of vision.

### 3. ****Decreased Visual Acuity****

- As cone cells degenerate, **central vision becomes affected.**
- Reading, recognizing faces, or doing fine visual tasks becomes harder.

### 4. ****Photopsia (Flashing Lights)****

- Some patients experience flashes of light, especially in the peripheral vision.

### 5. ****Color Vision Deficits****

- As cone function declines, **color discrimination becomes impaired**, particularly in the later stages.

### 6. ****Complete Blindness (in Severe Cases)****

- Some individuals may progress to total blindness, though this varies widely depending on the genetic type and progression rate.

Let me know if you'd like a diagram or if you're focusing on a specific age group or subtype of RP!

Can Retinitis Pigmentosa cause complete blindness?

Yes, **Retinitis Pigmentosa (RP)** can eventually lead to **complete blindness**, but this is not the typical course for most patients.

### Here's a breakdown:

- **RP is a group of inherited retinal dystrophies** that primarily affect the **photoreceptor cells** (rods first, then cones).
- **Early symptoms** usually include **night blindness** and **progressive loss of peripheral vision** (tunnel vision).
- Over time, **central vision can also deteriorate**, especially in advanced stages.

### In some cases:

- **Complete blindness** (no light perception) **can occur**, especially in severe forms or syndromic types (e.g., **Usher syndrome**).
- However, many individuals retain some **residual central vision** even in later stages.

### Prognosis depends on:

- **Genetic subtype** of RP
- **Onset age** and **rate of progression**
- Presence of **complications** like cataracts or macular edema

Would you like info on current treatments or visual rehabilitation options for advanced RP?

Can we stop Retinitis Pigmentosa from progressing?

As of now, **there’s no definitive cure** to stop the progression of **Retinitis Pigmentosa (RP)** entirely, but there have been **significant advancements** that can **slow it down**, **manage symptoms**, or **restore some degree of vision** in certain cases. Here's an overview of current strategies:

### 🔬 ****1. Gene Therapy****

- **Approved therapy**: Luxturna (voretigene neparvovec) for patients with **RPE65 mutations** (a specific form of RP).
- It delivers a normal copy of the faulty gene via a viral vector to retinal cells.
- It's the **first FDA-approved gene therapy** for a genetic eye disease and has shown promising results in **improving functional vision**.

### 💊 ****2. Nutritional Supplements****

- **Vitamin A palmitate** (15,000 IU/day) has been shown in some studies to **slow disease progression**, but:
  - Should be used with **caution** and under supervision.
  - Contraindicated in certain genetic types (like ABCA4 mutations).
- **Omega-3 fatty acids** and **lutein** may also support retinal health.

### 🧬 ****3. Ongoing Research****

- **CRISPR-based gene editing**: Experimental trials (e.g., EDIT-101 for CEP290 mutation).
- **Stem cell therapies**: Aim to replace damaged photoreceptors.
- **Retinal implants**: Like the Argus II (limited availability), providing some visual input in advanced cases.
- **Neuroprotective agents**: Trials are exploring molecules to prevent photoreceptor death.

### 👁️‍🗨️ ****4. Low Vision Rehabilitation****

- Even if we can’t stop progression entirely, **rehabilitation** (like magnifiers, orientation & mobility training, and assistive technology) can **greatly improve quality of life**.

### 🧬 Is RP always progressive?

Yes—RP is **progressive by nature**, but the **rate and pattern vary** depending on the **underlying gene mutation**. Some forms begin in childhood and progress rapidly, while others have a slower course.

If you're thinking of a patient or a research direction, I’d be happy to dive deeper into any specific subtype or therapeutic approach.

Can Retinitis Pigmentosa be reversed?

As of now, **Retinitis Pigmentosa (RP) cannot be reversed** in the traditional sense. It's a **genetic, progressive retinal dystrophy**, which means it typically worsens over time due to the degeneration of photoreceptor cells, primarily rods, and later cones.

However, there are some **emerging treatments and ongoing research** that offer hope for slowing progression or partially restoring vision in some cases:

### 🔬 ****Promising Approaches Under Study or In Limited Use****

1. **Gene Therapy**
   - Most effective in patients with **specific gene mutations** (e.g., RPE65).
   - **Luxturna** is an FDA-approved gene therapy for RPE65-related RP and Leber congenital amaurosis.
   - This doesn’t reverse damage already done, but **can preserve or slightly improve function** in some cases.
2. **Retinal Implants (Bionic Eyes)**
   - Devices like **Argus II** (limited use) aim to restore **basic visual perception** by bypassing damaged photoreceptors and stimulating the retina directly.
   - These offer **low-resolution vision**, often helping with light/dark perception and object outlines.
3. **Stem Cell Therapy**
   - Experimental; aims to **replace degenerated photoreceptors** or support surviving retinal cells.
   - Still under clinical trials—**not yet widely available or proven** for routine use.
4. **Optogenetics**
   - Converts remaining retinal cells into **light-sensitive cells** using gene therapy + external devices (like light-stimulating goggles).
   - Early clinical trials have shown **partial visual improvements**.
5. **Neuroprotective agents & Vitamin A**
   - Some evidence suggests **high-dose vitamin A palmitate** may slow progression in certain RP types, but it must be used cautiously due to liver toxicity risks.
   - Omega-3 supplements may have a **supportive role**.

### ✅ Summary

- **No cure or full reversal yet.**
- Early genetic diagnosis is crucial to determine eligibility for **emerging treatments**.
- Patients benefit from **low vision rehabilitation**, mobility training, and assistive devices.
- Clinical trials offer hope—**personalized genetic therapy may be the future**.

If you’re working on something related (like an article, presentation, or patient education), I can help tailor this further.

Can sunlight exposure worsen vision of people with Retinitis Pigmentosa?

Yes, **sunlight exposure can potentially worsen vision in people with Retinitis Pigmentosa (RP)** over time.

### Here's why:

1. **Phototoxicity risk**: The retina is already vulnerable in RP due to the degeneration of photoreceptors (mainly rods, then cones). Prolonged exposure to **ultraviolet (UV)** and **high-energy visible (HEV)** light (blue light) may accelerate oxidative stress and damage the remaining retinal cells.
2. **Scientific support**:
   - Studies have shown that **light-induced retinal damage** (particularly from UV and blue light) may worsen retinal degeneration in experimental models of RP.
   - Some observational studies suggest that **people with RP who protect their eyes from sunlight** (using sunglasses or UV/blue-blocking lenses) may have **slower progression** of the disease.
3. **Clinical recommendations**:
   - **UV-protective sunglasses** and **blue-blocking lenses** are often recommended, especially outdoors.
   - Wearing **hats with brims** and avoiding **bright light conditions** can also help reduce retinal stress.
   - **Photochromic lenses** or specially tinted filters (like amber or yellow lenses) may enhance contrast and comfort in RP patients while also offering protection.

### Bottom line:

While sunlight won’t directly cause RP, **chronic unprotected exposure may contribute to faster progression** due to added stress on the already degenerating retina. So protecting the eyes from bright light and UV/blue light is considered a prudent preventive measure.

Would you like references from the literature or tips on recommending protective eyewear to RP patients?

Can I treat Retinitis Pigmentosa by nutritional care?

Nutritional care **cannot cure or halt** the progression of **Retinitis Pigmentosa (RP)**, but certain nutritional strategies **may help slow degeneration** or **support overall retinal health** in some patients.

Here’s a breakdown of what the evidence says:

### 🔍 What Nutritional Strategies Have Been Studied?

#### 1. **Vitamin A Palmitate**

- **Some studies** (especially from the 1990s and early 2000s) suggested **high-dose Vitamin A (15,000 IU/day)** might slow progression in certain types of RP.
- But:
  - It's **not effective in all types** (especially not in Usher syndrome).
  - **High doses can be toxic** (liver, bone, teratogenic risks).
  - Long-term use requires **monitoring of liver function and vitamin levels**.

#### 2. **Omega-3 Fatty Acids (DHA)**

- Some evidence suggests diets **rich in DHA (e.g., from fish)** may help **enhance the effect of Vitamin A**.
- Still, results are mixed and **no definitive recommendation** exists.

#### 3. **Lutein and Zeaxanthin**

- These antioxidants accumulate in the retina and are beneficial for **macular health**, especially in age-related macular degeneration.
- Limited evidence for RP specifically, but may be helpful **as part of overall retinal health support**.

#### 4. **Other Antioxidants**

- Vitamins C, E, zinc, and selenium have been explored, but **no conclusive benefit** for RP.

### ⚠️ Important Notes

- Nutritional care should **not replace genetic counseling or low vision rehab**.
- Consider nutrition as a **supportive strategy**, not as a primary treatment.
- Always tailor to the **genetic subtype** of RP—some may respond differently.
- **Avoid megadoses** without medical supervision.

### ✅ Practical Takeaways

- A **balanced diet rich in antioxidants and omega-3s** can be helpful.
- **Vitamin A supplementation** may be considered in specific cases—but only under supervision.
- Patients should be advised to **avoid smoking**, which worsens oxidative stress.

If you're treating or counseling someone with RP, I can help create a patient-friendly nutrition guide or prepare a short evidence-based summary for colleagues—just let me know.

Is Retinitis Pigmentosa treatable?

Retinitis Pigmentosa (RP) currently has **no cure**, but there are **treatment options** that can **slow progression**, **manage symptoms**, or **help maintain functional vision**. The approach depends on the specific type of RP, the patient's symptoms, and genetic findings.

### Current treatment options:

#### 🧬 **Gene Therapy**

- **Luxturna (voretigene neparvovec)**: Approved for patients with **biallelic RPE65 mutations**. It’s the first FDA-approved gene therapy for inherited retinal disease.
- Other gene therapies are under investigation for different mutations (e.g., RPGR, USH2A).

#### 🧪 **Clinical Trials**

- Ongoing studies include **CRISPR-based gene editing**, **RNA therapy**, **stem cell therapy**, and **neuroprotective agents**.

#### 🌞 **Vitamin A Palmitate**

- Some evidence (Berson et al.) suggests **high-dose Vitamin A (15,000 IU/day)** may **slow progression** in certain types of RP.
- Must be used with caution—monitor liver function and avoid in women who may become pregnant.

#### 🕶️ **Low Vision Aids & Rehabilitation**

- **Magnifiers**, **CCTV**, **orientation and mobility training**, and **occupational therapy** help patients maximize their remaining vision.

#### 💡 **Retinal Implants (Bionic Eye)**

- Devices like the **Argus II** have been explored, but availability and effectiveness are limited.
- Research is ongoing.

#### 🧫 **Nutritional Supplements**

- **Omega-3 fatty acids**, **lutein**, **zeaxanthin**, and antioxidants are being studied, though data is less conclusive.

#### 🔍 **Genetic Testing & Counseling**

- Important for determining eligibility for gene therapies and understanding inheritance patterns.

Would you like a breakdown of current clinical trials or management options tailored for pediatric vs. adult patients?

What should I eat if I have Retinitis Pigmentosa?

If you have **Retinitis Pigmentosa (RP)**, a healthy diet won't cure or stop the disease, but it may support overall eye health and potentially slow progression in some cases. Here's a guide on what to eat, based on research and expert recommendations:

### 🥦 ****Best Foods for Retinitis Pigmentosa****

#### 1. **Foods Rich in Vitamin A (in moderation)**

- **Why:** Some studies suggest Vitamin A palmitate may slow RP progression.
- **Examples:**
  - Sweet potatoes
  - Carrots
  - Kale
  - Spinach
  - Butternut squash
- **Caution:** Only supplement under medical supervision — excess vitamin A can be toxic.

#### 2. **Omega-3 Fatty Acids**

- **Why:** May help slow vision loss, especially in combination with Vitamin A.
- **Examples:**
  - Salmon
  - Sardines
  - Chia seeds
  - Flaxseeds
  - Walnuts

#### 3. **Antioxidant-Rich Foods**

- **Why:** RP involves oxidative stress; antioxidants may offer protection.
- **Examples:**
  - Berries (blueberries, strawberries)
  - Leafy greens
  - Green tea
  - Broccoli
  - Bell peppers

#### 4. **Lutein and Zeaxanthin**

- **Why:** These carotenoids are concentrated in the retina and protect photoreceptors.
- **Examples:**
  - Egg yolks
  - Corn
  - Kale
  - Spinach
  - Orange peppers

#### 5. **Zinc and Selenium**

- **Why:** Important for retinal function and antioxidant enzyme activity.
- **Examples:**
  - Pumpkin seeds
  - Lentils
  - Brazil nuts (rich in selenium)
  - Oats
  - Whole grains

### 🥤 ****Stay Hydrated and Maintain General Health****

- Drink plenty of water
- Avoid smoking and limit alcohol
- Maintain a healthy weight and exercise regularly

### 🚫 ****Foods to Limit or Avoid****

- Processed foods high in sugar or trans fats
- Excess salt
- Fried and greasy foods
- Highly refined carbs (white bread, sugary cereals)

### 💊 Should You Take Supplements?

- **Vitamin A palmitate (15,000 IU/day)** has shown benefit in some RP patients (only with doctor approval).
- **DHA (Omega-3), lutein/zeaxanthin**, and **multivitamins** may be helpful.
- Always talk to your ophthalmologist or a low vision specialist before starting any supplements.

If you'd like, I can help you make a sample meal plan for RP-friendly eating too. Want one?

What foods should I avoid if I have Retinitis Pigmentosa?

If you have **Retinitis Pigmentosa (RP)**, there's no specific diet that can cure or halt the disease, but some dietary choices may help slow progression or support overall retinal health. Here are **foods to avoid or limit**, based on current understanding and recommendations:

### 🚫 Foods to Avoid or Limit

#### 1. **High-Sugar Foods**

- Excessive sugar can lead to oxidative stress and inflammation, both harmful to retinal cells.
- **Examples**: candies, pastries, sugary cereals, sodas, sweetened drinks.

#### 2. **Highly Processed Foods**

- Often high in trans fats, preservatives, and refined carbohydrates that can promote inflammation.
- **Examples**: fast food, frozen meals, chips, processed meats (sausages, salami), packaged snacks.

#### 3. **Fried and Greasy Foods**

- High in unhealthy fats (trans fats and some saturated fats) that may negatively impact retinal function.
- **Examples**: fried chicken, French fries, deep-fried snacks.

#### 4. **Excessive Omega-6 Fatty Acids**

- Too much omega-6 (without enough omega-3) can promote inflammation.
- **Examples**: corn oil, sunflower oil, soybean oil, margarine.

#### 5. **Alcohol (in excess)**

- Can deplete antioxidant levels (especially vitamin A and E) and contribute to oxidative stress.
- Occasional moderate drinking is usually fine, but excessive use is discouraged.

#### 6. **Very High Vitamin A (without supervision)**

- While some forms of vitamin A (like **vitamin A palmitate**) are used in controlled doses in RP, **excessive unsupervised intake** can be toxic.
- Avoid megadoses or vitamin A supplements unless prescribed by your ophthalmologist.

### ✅ Better Choices for Retinal Health

- **Leafy greens**: spinach, kale, rich in lutein/zeaxanthin
- **Omega-3 sources**: salmon, sardines, flaxseeds
- **Nuts & seeds**: walnuts, chia, almonds (vitamin E)
- **Bright-colored fruits/vegetables**: carrots, berries, oranges (antioxidants)
- **Whole grains**: brown rice, quinoa, oats

Would you like a sample meal plan or a list of supplements that may be helpful for RP (based on clinical studies)?

What is the prognosis for retinitis pigmentosa?

The **prognosis for retinitis pigmentosa (RP)** varies widely depending on the **genetic subtype**, **onset age**, and whether the condition is **syndromic or non-syndromic**, but here are some general points:

### 🔍 General Prognosis

- **Progressive vision loss**: RP is a slowly progressive degenerative disease. It typically starts with **night blindness** (nyctalopia) in adolescence or early adulthood, followed by **peripheral vision loss** (tunnel vision), and eventually may affect **central vision**.
- **Legal blindness**: Many patients with RP become legally blind by **age 40–50**, although they may retain some central vision for reading or recognizing faces even later.

### 🧬 Genetic Influence

- **Autosomal dominant RP**: Tends to have the **best prognosis**, with slower progression.
- **Autosomal recessive RP**: Intermediate severity.
- **X-linked RP**: Often has the **worst prognosis**, with earlier onset and more rapid progression.

### 👁 Central Vision & Macula

- Central vision often remains relatively good until later stages, but in some cases, **macular edema**, **epiretinal membrane**, or **cataracts** (especially posterior subcapsular) can worsen central vision earlier.

### 🧑‍⚕️ Syndromic Forms

- Prognosis can be more complex in syndromic RP, such as:
  - **Usher syndrome**: RP with hearing loss. Prognosis is affected by both sensory impairments.
  - **Bardet-Biedl syndrome**: Often more severe vision loss due to retinal dystrophy and associated systemic findings.

### 📈 Advances and Monitoring

- **Genetic testing** helps with predicting prognosis and eligibility for **emerging gene therapies** (like voretigene neparvovec for RPE65-related RP).
- **Regular monitoring** with visual fields, OCT, and ERG helps assess progression and manage complications.

Would you like a quick table summarizing types and prognosis or info on current treatment options and clinical trials?

Can people with retinitis pigmentosa drive?

Whether people with **retinitis pigmentosa (RP)** can drive depends on the **severity and progression** of their visual impairment and the **driving laws** of their country or state. Here's a breakdown:

### ✅ ****In early stages****

- **Some individuals can legally drive**, especially if:
  - Their **central visual acuity** is good (e.g., 20/40 or better with correction),
  - They still have **adequate visual fields**, typically a minimum of 120° horizontal field (varies by region).
- They may require **frequent re-evaluation** of their vision.

### ⚠️ ****As RP progresses****

- **Peripheral vision loss** (tunnel vision) becomes more prominent.
- **Night blindness** (nyctalopia) makes driving at dusk or night dangerous.
- Difficulty with **glare, contrast sensitivity**, and **adapting to light changes** becomes more limiting.
- Many people with RP eventually **stop driving voluntarily** or are **legally disqualified** as vision drops below legal requirements.

### 🚗 ****Adaptive solutions (in some places)****

- **Bioptic telescopic glasses**: Some jurisdictions allow driving with bioptics if vision meets criteria.
- **Restricted licenses**: Daylight-only driving or local-area driving might be permitted in early stages.

### 🛑 ****In advanced stages****

- When peripheral vision drops below 20°, or visual acuity drops below 20/70 (varies), driving is generally **no longer legal or safe**.

If you're considering driving with RP (or advising someone who is), the best steps are:

1. **Consult an ophthalmologist** for updated visual field and acuity testing.
2. **Check local driving laws** or medical driving standards.
3. Consider **occupational therapy driving evaluations** or low vision driving programs if borderline.

Want me to summarize the driving standards in a specific country or state?

Does pregnancy have an effect on retinitis pigmentosa?

Pregnancy can have **some effects on retinitis pigmentosa (RP)**, though the data is limited and often anecdotal due to the rarity of the condition and variability among patients. Here's what is known:

### 1. ****Subjective Visual Changes****

- Some women with RP report **worsening of visual symptoms during pregnancy**, such as:
  - Increased night blindness
  - Decreased visual field
  - More glare or photophobia
- These changes are often **transient** and may return to baseline after delivery.

### 2. ****Possible Mechanisms****

- Hormonal fluctuations (especially estrogen and progesterone) during pregnancy may affect the **retinal pigment epithelium (RPE)** or **retinal metabolism**.
- Pregnancy-related **vascular or fluid shifts** could also influence retinal function.
- However, there's **no strong evidence** of permanent structural progression directly due to pregnancy.

### 3. ****Electrophysiological Findings****

- Some studies using **electroretinography (ERG)** have shown mild decreases in response during pregnancy, but again, these changes often normalize postpartum.

### 4. ****Genetic Considerations****

- Since RP is often **inherited**, genetic counseling is important during pregnancy.
- Options like **prenatal diagnosis** or **preimplantation genetic testing (PGT)** may be discussed for families with known mutations.

### 5. ****Clinical Recommendations****

- Regular follow-up during pregnancy is reasonable, especially if the patient reports subjective worsening.
- Most cases do not require any change in management unless there’s a significant or unexpected decline.

In summary: **Pregnancy may cause mild, usually temporary changes in visual function in women with RP**, but does not appear to accelerate long-term disease progression based on current evidence.

Would you like references or case study examples for a presentation or paper?

What is the likelihood that I will pass the retinitis pigmentosa gene on to my children?

The likelihood of passing **retinitis pigmentosa (RP)** to your children depends on the **specific genetic inheritance pattern** of the RP in your family. RP can be inherited in several ways:

### 1. ****Autosomal Recessive (AR) RP****

- **Most common form.**
- Both parents must be carriers of the faulty gene.
- If **you have AR RP** (i.e., two copies of the mutated gene), your children will **inherit one mutated gene** but usually will **not be affected**—they will be **carriers**.
- If your partner is also a carrier, there's a:
  - **25% chance** the child will have RP
  - **50% chance** they’ll be a carrier
  - **25% chance** they’ll be unaffected

### 2. ****Autosomal Dominant (AD) RP****

- Only **one copy** of the mutated gene is needed to cause RP.
- If **you have AD RP**, there’s a:
  - **50% chance** you will pass the disease-causing gene to each child
  - That child will likely develop RP

### 3. ****X-linked RP****

- Gene is located on the X chromosome.
- If **you are male and affected**, **all your daughters** will be carriers, but **none of your sons** will inherit it.
- If **you are female and a carrier**, there's a:
  - **50% chance** each **son** will be affected
  - **50% chance** each **daughter** will be a carrier

To know **your specific risk**, it's important to:

✅ **Identify the inheritance pattern** in your family
✅ **Consult a genetic counselor**
✅ Consider **genetic testing** to determine the specific mutation

Would you like help understanding a specific family history or scenario?

What should I do if my child is diagnosed with retinitis pigmentosa?

If your child has been diagnosed with **retinitis pigmentosa (RP)**, it’s natural to feel overwhelmed. While there's no cure yet, there are important steps you can take to support your child’s vision, development, and emotional well-being. Here’s a guide to help you navigate this journey:

### 🧠 ****Understand the Diagnosis****

- **RP is a progressive retinal disorder** that affects night vision and peripheral vision first, and can eventually impact central vision.
- It’s often **genetic**, so it may be helpful to undergo **genetic counseling** to understand inheritance patterns and risks for other family members.

### 👨‍⚕️ ****Assemble a Care Team****

- **Pediatric Ophthalmologist / Retina Specialist** – for regular monitoring and treatment.
- **Low Vision Specialist** – to evaluate functional vision and provide visual aids.
- **Genetic Counselor** – to explain the genetic cause and potential clinical trials.
- **Occupational Therapist / Orientation & Mobility Specialist** – to help with adapting daily activities and navigating safely.

### 🧑‍🏫 ****Educational Support****

- Notify your child’s school. They may qualify for:
  - **Individualized Education Plan (IEP)** or **504 Plan**
  - Assistive technology (e.g., screen readers, magnifiers)
  - Modified classroom lighting and materials
- Vision teachers can help your child learn **Braille** if needed, and build skills for independence.

### 🧪 ****Explore Treatment & Research****

- There’s no cure yet, but clinical trials are ongoing, especially in **gene therapy**.
- Ask your doctor if your child qualifies for:
  - **Gene therapy** (e.g., Luxturna is approved for some types of RP)
  - **Vitamin A** supplementation (only under medical supervision)
  - **Clinical research participation** through centers or patient registries

### 🧰 ****Maximize Remaining Vision****

- Use **low vision aids**: handheld magnifiers, electronic devices, special glasses.
- Ensure good **lighting and contrast** at home and school.
- Avoid eye injuries—wear protective glasses during sports or risky activities.

### ❤️ ****Support Your Child Emotionally****

- Children with RP may feel isolated or anxious about their future.
- Encourage open conversations about their feelings and needs.
- Connect with **support groups** (e.g., Foundation Fighting Blindness) and other families.
- Celebrate their strengths and interests—RP doesn’t define who they are.

### 🏡 ****Plan for Independence and the Future****

- Teach **life skills early**: mobility, self-advocacy, and using assistive tech.
- Focus on abilities and resilience.
- Career planning should start early—with accommodations, people with RP can thrive in diverse fields.

If you’d like, I can help you find local resources or support groups, or guide you on how to prepare your child for school and social life with low vision. Would that be helpful?

What is the extent of independence for people with advanced retinitis pigmentosa?

The extent of independence for people with **advanced retinitis pigmentosa (RP)** varies widely depending on several factors, including the **severity and progression of vision loss**, **access to assistive technology**, **rehabilitation services**, and **individual adaptability**. Here’s a breakdown of what to expect:

### ✅ ****What’s Typically Affected in Advanced RP****

- **Peripheral vision** is severely constricted, often resulting in **tunnel vision**.
- In late stages, **central vision** may also deteriorate, impacting detailed tasks.
- **Night blindness** and difficulty adapting to changes in lighting are common.
- Depth perception and contrast sensitivity may be reduced.

### 🧭 ****Daily Independence – What’s Possible?****

#### 1. **Mobility**

- With training (e.g., orientation & mobility training), individuals can navigate using:
  - **White canes**
  - **Guide dogs**
  - **GPS-based navigation apps**
- Environments that are **structured and familiar** are easier to manage.

#### 2. **Reading and Writing**

- Many use **screen readers**, **braille displays**, or **magnification tools**.
- Audiobooks and text-to-speech tools like **VoiceOver** or **JAWS** enhance access.

#### 3. **Communication and Technology**

- Smartphones, tablets, and computers can be used independently via:
  - **Voice commands**
  - **Haptic feedback**
  - **Accessibility settings** (zoom, contrast, screen reading)

#### 4. **Employment**

- Some individuals with advanced RP work in jobs suited to their skills and assisted by technology, such as:
  - Counseling, teaching, programming, transcription, or voice acting
- Success is highly dependent on **workplace accessibility** and **supportive policies**.

#### 5. **Self-Care and Home Life**

- With adaptations (e.g., tactile markings, voice-controlled devices), most can:
  - Cook with safety tools
  - Handle personal care
  - Use public transportation (with support in unfamiliar areas)

### 🔧 ****Supports That Boost Independence****

- **Low vision rehabilitation**
- **Occupational therapy**
- **Assistive tech training**
- **Peer support or counseling**
- **Social support systems** (family, community resources)

### ⚠️ ****Challenges****

- Risk of **falls and injuries**
- **Social isolation** if mobility and social participation decline
- **Mental health** concerns (e.g., anxiety, depression) if not addressed

### 💡 Summary

People with advanced RP **can maintain a significant degree of independence**, especially with access to **training, assistive tools, and a supportive environment**. The key is **early intervention**, **personal adaptation**, and **ongoing support**.

Would you like this in a visual format (e.g., chart or infographic) or tailored for a particular audience like patients, clinicians, or students?

What part of the eye is affected by retinitis pigmentosa?

Retinitis pigmentosa (RP) primarily affects the **retina**, specifically the **photoreceptor cells**—**rods** and **cones**.

- **Rods**, which are responsible for night vision and peripheral vision, are usually affected first. This leads to **night blindness** and **tunnel vision**.
- As the disease progresses, **cones**, which are responsible for central and color vision, can also degenerate, causing **central vision loss**.

Over time, this degeneration of photoreceptors can lead to **severe vision impairment or blindness**.

Let me know if you want a diagram or more detail on the retinal layers involved.

Can women get retinitis pigmentosa?

Yes, women **can** get retinitis pigmentosa (RP).

RP is a group of inherited retinal dystrophies, and the inheritance pattern determines how it affects men and women:

### Common inheritance patterns:

1. **Autosomal recessive** – affects both males and females equally.
2. **Autosomal dominant** – affects both males and females equally.
3. **X-linked recessive** – more severe in males, but **females can still be affected**, especially if they are:
   - **Carriers** with skewed X-inactivation (some retinal cells express the mutated gene),
   - Or **homozygous** in rare cases (e.g., consanguinity).

### In summary:

- Yes, women can definitely develop RP.
- Severity may vary depending on the inheritance pattern.
- Genetic testing can clarify the type and risk in families.

If you’re looking at it from a clinical, academic, or research perspective, I can go deeper into specific genes or phenotypes—just let me know!

How long is the average lifespan of a person with retinitis pigmentosa?

Retinitis pigmentosa (RP) primarily affects vision, not general health or lifespan. Most people with RP have a **normal life expectancy**.

However, the quality of life can be significantly impacted due to progressive vision loss, typically starting with night blindness and peripheral vision loss, eventually leading to tunnel vision or complete blindness in severe cases. The **rate and extent of vision loss** vary greatly depending on the genetic type of RP and whether it's part of a syndrome (like Usher or Bardet-Biedl syndromes).

In **syndromic forms** of RP (e.g., Bardet-Biedl syndrome), lifespan may be affected by associated systemic issues like obesity, kidney disease, or heart problems. But in **non-syndromic RP**, there's no inherent reduction in life expectancy.

Let me know if you want info on specific types or genetic subtypes of RP.

Is retinitis pigmentosa a disability?

Yes, **retinitis pigmentosa (RP)** is considered a **disability** in many contexts because it is a **progressive degenerative eye disease** that can lead to **significant vision loss or blindness** over time.

### Why RP is considered a disability:

- **Legal definitions** of disability in many countries (e.g., under the Americans with Disabilities Act or the UN Convention on the Rights of Persons with Disabilities) include visual impairments that substantially limit one or more major life activities, such as seeing.
- RP often causes:
  - **Night blindness**
  - **Tunnel vision (loss of peripheral vision)**
  - **Severe vision loss or blindness** in advanced stages
- People with RP often require:
  - **Visual aids** or **low vision rehabilitation**
  - **Occupational adjustments**
  - **Mobility training (e.g., white cane, guide dog)**

### Recognition and Support:

- Individuals with RP may be eligible for:
  - **Disability benefits**
  - **Assistive technology and support services**
  - **Educational accommodations**
  - **Workplace accommodations**

If you're asking this in relation to a medical, legal, or social service context (e.g., for documentation or research), I can help tailor the explanation accordingly.

What does a person with retinitis pigmentosa see?

A person with **retinitis pigmentosa (RP)** typically experiences a gradual and progressive loss of vision due to the degeneration of photoreceptor cells—primarily **rods**, which are responsible for peripheral and night vision, and later **cones**, which are responsible for central and color vision.

### Common Visual Experiences in RP:

#### 1. **Night Blindness (Nyctalopia):**

- One of the earliest symptoms.
- Difficulty seeing in dim or dark environments (e.g., theaters, night driving).

#### 2. **Tunnel Vision:**

- Gradual loss of **peripheral (side) vision**, leading to a narrowing of the visual field.
- Feels like looking through a tunnel or a narrow tube.
- The central vision is often preserved until late stages.

#### 3. **Photopsia (Flashing Lights):**

- Seeing flickering or shimmering lights, especially in dark conditions.

#### 4. **Glare Sensitivity:**

- Difficulty adapting to bright light or transitioning between different lighting environments.

#### 5. **Later Stages – Central Vision Loss:**

- As cones become affected, reading, recognizing faces, and detailed tasks become difficult.
- In advanced stages, vision may reduce to **light perception or total blindness**.

### Visual Simulation (Descriptive):

Imagine walking through a hallway:

- At first, the sides slowly fade away.
- You can see directly ahead but are unaware of people or objects beside you.
- Over time, even the central area becomes dim, as if looking through a fogged keyhole.
- In low light, you may barely see anything at all.

Would you like me to generate a visual simulation image of how someone with RP might see in different stages?

How quickly does retinitis pigmentosa progress?

The progression of **retinitis pigmentosa (RP)** varies widely depending on the **genetic subtype**, but in general, it is a **slowly progressive disease** that typically worsens over **decades**.

Here's a general breakdown:

### 👶 Onset

- Usually starts in **childhood or adolescence**, but some forms begin in **early adulthood** or even **later**.

### 🧭 Progression Timeline

- **Night blindness** (nyctalopia) is often the **first symptom**, appearing in childhood or teens.
- **Peripheral vision loss** (tunnel vision) develops slowly over the years.
- **Central vision** is usually preserved until **late stages**—often into the **4th to 5th decade of life**.
- **Legal blindness** (visual acuity <20/200 or visual field <20°) may occur by the **40s or 50s**, but this can vary.

### 📉 Factors Affecting Progression

- **Genetic type**:
  - **Autosomal dominant RP** tends to be milder and slower.
  - **Autosomal recessive** and **X-linked RP** forms progress faster.
- **Syndromic RP** (like Usher syndrome or Bardet-Biedl) often involves **faster visual deterioration** due to associated systemic problems.
- **Environmental and lifestyle factors** (light exposure, smoking, etc.) might play a minor role, though data is limited.

### 🧬 Modern Updates

- Genetic testing helps predict progression more accurately.
- Emerging treatments (like gene therapy or retinal implants) are being developed, especially for early-to-mid-stage RP.

Would you like a chart comparing progression timelines between different RP types or syndromic vs non-syndromic forms?

What if a person suffering from retinitis pigmentosa develops cataract?

If a person with **retinitis pigmentosa (RP)** develops a **cataract**, it can significantly affect their remaining vision, but there are also important considerations for management. Here's a breakdown:

### 🔍 Why is this important?

People with RP already have **progressive degeneration of the retina**, especially affecting peripheral vision and night vision. Adding a **cataract**—particularly a **posterior subcapsular cataract (PSC)**, which is common in RP—can further reduce central vision and contrast sensitivity.

### 👁️ Common Effects of Cataract in RP:

- **Further blurred vision** (especially for near tasks)
- **Glare and light sensitivity** (often more pronounced)
- **Reduced contrast sensitivity**
- A feeling of “foggy” or “hazy” vision

### 🩺 Management Considerations:

#### 1. **Cataract Surgery is Often Beneficial**

- Many RP patients benefit from cataract removal, especially if they still have usable central vision.
- Studies show significant subjective improvement in **quality of life** and **visual function**, even if peripheral vision remains limited.

#### 2. **Preoperative Evaluation is Key**

- Careful **retinal assessment** (OCT, ERG) to ensure the patient’s vision loss is at least partly due to the cataract.
- Consider **visual potential**—if the macula is relatively preserved, results are more favorable.

#### 3. **Surgical Considerations**

- Standard phacoemulsification with intraocular lens (IOL) implantation is used.
- **Capsular fragility** may be more common due to zonular weakness.
- Choice of IOL: Avoid multifocal lenses (poor contrast sensitivity); monofocal or enhanced-depth-of-focus lenses may be better.

#### 4. **Postoperative Expectations**

- Vision may improve, particularly **central acuity and contrast**.
- Peripheral field and night vision deficits from RP will not be reversed.
- Patients must have **realistic expectations**.

### ✅ Summary:

Cataract surgery can significantly **improve the quality of life** in RP patients, especially when performed thoughtfully. It won't stop the progression of RP but can help maximize existing vision.

If you're dealing with a specific patient scenario or study focus related to this, I can help tailor the advice more closely—just let me know!

Does "night-blindness" mean a person has retinitis pigmentosa?

Not necessarily. While **night-blindness** (nyctalopia) is a common **early symptom of retinitis pigmentosa (RP)**, it **doesn’t automatically mean** someone has RP. Night-blindness simply refers to **difficulty seeing in low light or darkness**, and it can be caused by a variety of conditions.

### Common Causes of Night-Blindness:

1. **Retinitis Pigmentosa (RP)** – progressive genetic disorder that leads to degeneration of the photoreceptors, especially rods (which are responsible for night vision).
2. **Vitamin A deficiency** – affects the function of photoreceptors; common in malnutrition or fat malabsorption syndromes.
3. **Congenital stationary night blindness (CSNB)** – a non-progressive inherited disorder.
4. **Cataracts** – especially nuclear sclerosis, which can scatter light and worsen night vision.
5. **Myopia** – high degrees of nearsightedness can reduce contrast sensitivity in low light.
6. **Certain medications** – e.g., phenothiazines or chloroquine may have retinal side effects.
7. **Liver disease** – can interfere with vitamin A metabolism.

### So how do you tell if it's RP?

- **Progressive loss of peripheral vision** (tunnel vision) over time.
- **Bone-spicule pigmentation** seen on fundus exam.
- **Abnormal ERG (electroretinogram)** showing reduced rod response.
- **Family history** and genetic testing support diagnosis.

In summary: **Night-blindness is a red flag** that warrants further investigation but isn't diagnostic of RP on its own.

Which doctor should you consult for retinitis pigmentosa?

For **retinitis pigmentosa (RP)**, you should consult an **ophthalmologist**, specifically one with expertise in:

1. **Retina specialist (vitreoretinal specialist)** – They focus on diseases of the retina and are best suited for diagnosing and managing RP. They can monitor disease progression, manage complications (like cataracts or macular edema), and discuss emerging treatments (e.g., gene therapy, clinical trials).
2. **Low vision specialist** – Often an optometrist or ophthalmologist with training in low vision rehabilitation. They can help maximize remaining vision with assistive devices and strategies.
3. **Genetic counselor or medical geneticist** – Because RP is usually inherited, a genetic consultation can help with diagnosis, family planning, and eligibility for gene-specific therapies or trials.

Let me know if you want to know about specific treatments or centers that specialize in RP.
