# Supplementary material for: Evaluation of the artificial intelligence chatbots in frequently asked questions about retinitis pigmentosa: a comparative analysis between ChatGPT-4 and Gemini-2.0
Source: Int J Retina Vitreous. 2025 Nov 28;12:1. doi: 10.1186/s40942-025-00772-4 (PMC12763912; doi:10.1186/s40942-025-00772-4)
Supplement: Supplementary file 2 — Supplementary Material 2 [file 40942_2025_772_MOESM2_ESM.docx]

What is Retinitis Pigmentosa?

**Retinitis Pigmentosa (RP)** is an eye disease that people are usually born with. It slowly damages the part of the eye called the **retina**, which helps us see light, color, and details.

**What Happens in RP:**

- People often have trouble seeing at night.
- Over time, they lose **side vision**, so it’s like looking through a tunnel.
- Some people also lose their sharp, **central vision** later on.

**What Causes It:**

- It is caused by changes in **genes**, which are passed down from parents.
- It can run in families in different ways.

**How It’s Found:**

- Doctors use special tests to check how well the eyes respond to light.
- They might also look at pictures of the retina and do a **genetic test**.

**Can It Be Treated?**

- There is **no cure** yet, but doctors can help people make the most of their vision.
- Special glasses or tools can help with daily tasks.
- Some new treatments, like **gene therapy**, are being used for certain types.

Would you like a version with pictures or diagrams to help explain it further?

What causes you to get Retinitis Pigmentosa?

Retinitis Pigmentosa (RP) is an eye disease that people are usually **born with** because of a **problem in their genes**. Genes are like instructions in our bodies that tell our cells what to do. In RP, some of those instructions are broken, so the **cells in the eye that help us see—called photoreceptors—don’t work right** and slowly stop working over time.

These cells normally help you see in the dark and see things on the side (peripheral vision). As RP gets worse, it can also make it hard to see in bright light or to see details in front of you.

Most people with RP **get it from their parents**, even if no one else in the family has it. Sometimes, the gene changes happen by chance.

There’s no cure yet, but eye doctors can help protect vision and suggest tools or services to make seeing easier.

Let me know if you’d like this explained as a handout or a slide for patients or families.

Can Retinitis Pigmentosa be passed down through heredity?

Yes, Retinitis Pigmentosa (RP) can be passed down from parents to children. It is an eye disease that people are usually born with, even if it doesn’t cause vision problems right away.

RP happens because of changes (mutations) in certain genes. These genes can be passed down in different ways:

- **Sometimes, one parent has the gene and passes it to the child.**
- **Other times, both parents carry the gene without knowing it, and the child gets both copies.**
- **In some cases, the gene comes from the mother and mostly affects boys.**

Even if no one else in the family has RP, it can still show up in a child because of a new gene change.

Doctors can do genetic testing to learn more about the type of RP and how it might affect other family members.

Can Retinitis Pigmentosa be caused by injury or accident?

No, Retinitis Pigmentosa (RP) is not caused by an injury or accident. It is a condition you are born with because of changes (mutations) in your genes. These gene changes are usually passed down from parents to children.

RP affects the back part of your eye called the retina. Over time, it causes problems with your vision, like trouble seeing at night and loss of side vision.

Even if someone hurts their eye in an accident, it won’t cause RP. Injuries can damage the eye, but that’s a different kind of problem, not the same as Retinitis Pigmentosa.

What are the symptoms of Retinitis Pigmentosa?

Sure! Here's a simplified version of the symptoms of Retinitis Pigmentosa (RP) for a general audience:

**Retinitis Pigmentosa (RP)** is an eye disease that causes slow vision loss over time. It happens because the light-sensing cells in the back of the eye (called the retina) stop working properly.

Here are the common symptoms:

1. **Trouble seeing at night**
   - It becomes hard to see in the dark or in dim places, like movie theaters.
2. **Loss of side vision (tunnel vision)**
   - You may bump into things because you can’t see to the sides. It feels like you’re looking through a narrow tube.
3. **Flashes of light**
   - You might see little flashes or flickers of light that aren't really there.
4. **Being bothered by bright lights**
   - Sunlight or bright indoor lights can hurt your eyes or make it hard to see.
5. **Problems seeing colors**
   - Over time, colors may look faded or dull.
6. **Blurry central vision**
   - In later stages, it gets harder to read, recognize faces, or see things clearly in the center of your vision.

RP usually gets worse slowly, and it can lead to major vision loss over many years. There's no cure yet, but some treatments and tools can help people with RP live better and more safely.

How can Retinitis Pigmentosa be diagnosed?

Sure! Here’s a simple explanation of how **Retinitis Pigmentosa (RP)** is diagnosed, written at a sixth-grade reading level:

**How Do Doctors Know If Someone Has Retinitis Pigmentosa?**

Retinitis Pigmentosa (RP) is an eye disease that makes it hard to see, especially at night or in the dark. Over time, it can also make it hard to see from the sides, like looking through a tunnel.

Here’s how doctors find out if someone has RP:

**1. Talking About Symptoms**

The doctor will ask if you:

- Have trouble seeing at night.
- Bump into things because you can’t see from the sides.
- Have other family members with similar vision problems.

**2. Looking Into Your Eyes**

The doctor looks at the back of your eyes with a special light. They might see:

- Dark spots (like tiny clumps of pigment).
- Thin blood vessels.
- A pale optic nerve (the part that sends signals to your brain).

**3. Testing Your Side Vision**

You’ll look into a machine and press a button when you see little lights. This helps the doctor check how well you see around the edges.

**4. Measuring Eye Signals**

Doctors use a test called **ERG** (electroretinogram). It checks how your eyes respond to light. If the signals are weak or missing, it may mean you have RP.

**5. Taking Eye Pictures**

Using machines like **OCT** or **FAF**, doctors take pictures of the layers in your eyes. These pictures show if parts of your eye are getting thinner or damaged.

**6. Genetic Testing**

Sometimes, RP is passed down in families. A simple test using your spit or blood can show if you have a gene that causes RP.

This helps doctors know for sure if you have RP and how to plan your care.

How does Retinitis Pigmentosa affect vision?

Retinitis Pigmentosa (RP) is an eye disease that slowly causes vision loss over time. It happens because the cells in the back of the eye (called the retina) stop working properly.

Here’s how RP affects vision:

1. **Hard to See at Night**
   One of the first problems is trouble seeing in the dark or in low light. This is called night blindness.
2. **Loss of Side Vision**
   People with RP often lose their side vision first. This makes it feel like they are looking through a tunnel—called "tunnel vision."
3. **Blurry Central Vision**
   As the disease gets worse, it can make it hard to see things clearly in the center, like reading or seeing someone’s face.
4. **Flashing Lights**
   Some people may see flashing lights that aren’t really there.
5. **Trouble Seeing Colors**
   Over time, it might also get harder to tell colors apart.

RP usually gets worse slowly, and how fast it changes can be different for each person. Let me know if you’d like a drawing or simple chart to go with this!

Can Retinitis Pigmentosa cause complete blindness?

Yes, **Retinitis Pigmentosa (RP)** can cause **complete blindness**, but this doesn't happen to everyone.

RP is an eye disease that people are usually born with. It causes the light-sensing cells in the eye to slowly stop working. First, it becomes hard to see at night. Then, people slowly lose their side vision, like looking through a tunnel. Over many years, some people may lose all of their sight, but others still keep some central vision.

How fast it gets worse can be different for each person. There’s no cure yet, but there are tools and training that can help people with low vision live more easily.

Can we stop Retinitis Pigmentosa from progressing?

Sure! Here's a simpler explanation:

**Can we stop Retinitis Pigmentosa (RP) from getting worse?**

Right now, **we can't completely stop RP**, but doctors and scientists are working hard to find ways to **slow it down** or **help people see better**.

**Here’s what we have so far:**

**1. Gene therapy:**
There’s a special treatment called **Luxturna** that helps people with a certain kind of RP caused by a gene problem called **RPE65**. It can **improve vision** in some people.

**2. Vitamins:**
Some doctors may suggest taking **Vitamin A** and **fish oil (Omega-3)** to help slow down the disease. But you should always talk to your eye doctor first, because it’s not safe for everyone.

**3. New research:**
Scientists are testing new treatments like:

- Changing bad genes (gene editing),
- Using special cells to fix the eye (stem cells),
- And even eye implants to bring back some vision.

**4. Vision tools:**
Even if vision gets worse, there are **special tools and training** that help people with RP live more independently. These include magnifiers, talking devices, and special glasses.

So, we **can’t cure RP yet**, but we can help people with RP **see better for longer** and **live full lives**. There’s also a lot of **hope for the future**, thanks to new research.

Would you like this in flyer format or as a short video script?

Can Retinitis Pigmentosa be reversed?

Retinitis Pigmentosa (RP) is an eye disease that slowly causes vision loss over time. Right now, **there is no cure** and it **can’t be reversed**.

But scientists are working on new treatments that may help some people. For example:

- **Gene therapy** can help people with certain types of RP by fixing the broken gene. It may stop vision from getting worse.
- **Bionic eyes** (retinal implants) can help some people see shapes or lights again, but not full vision.
- **Stem cells and other new treatments** are being tested to try and fix the damaged parts of the eye.
- **Vitamin A** may help slow down vision loss in some people, but it’s not a cure and must be used carefully.

Even though RP can’t be reversed right now, people with RP can get help from **low vision tools** like special glasses, magnifiers, and training to live more independently.

Doctors and scientists are hopeful that better treatments will be available in the future.

Can sunlight exposure worsen vision of people with Retinitis Pigmentosa?

Sure! Here's a simple explanation:

Yes, being in the sunlight too much **can make vision worse** for people with **Retinitis Pigmentosa (RP)**.

RP is an eye disease that slowly causes people to lose their sight. The cells in the eye that help you see—especially in the dark—start to die. Sunlight, especially the strong kind with **UV rays** and **blue light**, can **hurt the eyes even more** and make the disease **get worse faster**.

**Here's what helps:**

- **Wearing sunglasses** that block UV and blue light when outside.
- **Wearing a hat** with a brim to block the sun.
- Using **special lenses** that change color in sunlight or help reduce glare.

These simple steps can help protect the eyes and **slow down the damage**.

Let me know if you'd like help picking out good sunglasses or explaining this to a patient or their family!

Can I treat Retinitis Pigmentosa by nutritional care?

Sure! Here's a version written simply for the average American adult at about a sixth-grade reading level:

**Can You Treat Retinitis Pigmentosa (RP) with Nutrition?**

Retinitis Pigmentosa (RP) is an eye disease that gets worse over time. It affects how well you can see, especially at night or in the dark. Right now, there is **no cure** for RP. But some vitamins and healthy foods **might help slow it down** a little for some people.

**What Might Help?**

**1. Vitamin A (Palmitate form):**

- Taking a certain kind of Vitamin A may help slow vision loss in some people with RP.
- But taking too much can **hurt your liver**, your bones, or even your baby if you are pregnant.
- Never take it unless your eye doctor tells you it's okay and checks your health.

**2. Omega-3 (from fish like salmon):**

- Eating fish a few times a week might help your eyes stay healthy.
- Omega-3 fats are good for your body and may work better if you also take Vitamin A (with a doctor’s help).

**3. Leafy greens and colorful veggies:**

- Foods like spinach, kale, and carrots have nutrients like lutein and zeaxanthin.
- These help protect your eyes from damage, but they don’t cure RP.

**What Doesn’t Work?**

- There is **no special diet or vitamin that cures RP**.
- Some other vitamins (like C, E, or zinc) may be good for general health but **won’t stop RP**.

**What Should You Do?**

- Eat a **healthy, balanced diet** with fruits, veggies, and fish.
- **Don’t smoke**—it’s bad for your eyes.
- Talk to your eye doctor before taking any vitamins or supplements.
- Get regular eye checkups and ask about **low vision tools** to help you see better.

If you want, I can also make this into a one-page handout for patients—just say the word!

Is Retinitis Pigmentosa treatable?

Retinitis Pigmentosa (RP) is an eye disease that causes vision to get worse over time. Right now, there is **no cure**, but there are ways to help people with RP **see better for longer**.

Here are some things that can help:

1. **Gene therapy** – For some people with a certain gene problem, a special treatment called **Luxturna** can help improve vision. It works best if used early.
2. **Vitamin A** – Some doctors may suggest **vitamin A** to slow down the vision loss. But it’s not safe for everyone, so a doctor should decide.
3. **Vision tools** – Things like **special glasses**, **magnifiers**, or **talking devices** can help people use the vision they still have.
4. **Training and support** – People with RP can learn new ways to get around safely and do daily tasks. This is called **low vision rehab**.
5. **New research** – Scientists are working on new treatments, like **gene editing**, **stem cells**, and **bionic eyes**, but most are still being tested.

If someone has RP, it’s important to see an **eye doctor** regularly and ask about **genetic testing** to see if special treatments might help.

What should I eat if I have Retinitis Pigmentosa?

Sure! Here's a simple and easy-to-understand answer about what to eat if you have **Retinitis Pigmentosa (RP)**:

If you have **Retinitis Pigmentosa**, eating healthy foods may help your eyes stay healthier for longer. It won’t cure the disease, but it can support your vision.

**Good Foods for Your Eyes:**

**1. Orange and green veggies**

- Carrots, sweet potatoes, spinach, and kale
- These have **vitamin A**, which may help slow down RP

**2. Fish**

- Salmon, sardines, and tuna
- These have **omega-3 fats**, which are good for your eyes

**3. Colorful fruits**

- Blueberries, strawberries, oranges
- These are full of **antioxidants**, which protect your eyes

**4. Eggs and corn**

- These have nutrients called **lutein** and **zeaxanthin**
- They help protect your retina (the part of your eye that helps you see)

**5. Nuts and seeds**

- Walnuts, flaxseeds, and pumpkin seeds
- They give your body healthy fats and minerals like **zinc**

**Foods to Avoid or Eat Less Of:**

- Fried foods
- Sugary snacks and sodas
- Salty chips
- White bread and candy

These can be bad for your eyes and your health in general.

**Should You Take Vitamins?**

Some people with RP take **vitamin A supplements**. But too much can be harmful, so **ask your eye doctor first**.

**Other Tips:**

- Drink plenty of water
- Don’t smoke
- Exercise and get enough sleep

Would you like a simple meal plan too?

What foods should I avoid if I have Retinitis Pigmentosa?

Sure! Here's a simple explanation just right for a sixth-grade reading level:

If you have **Retinitis Pigmentosa (RP)**, eating the right foods might help keep your eyes as healthy as possible. While food can't cure RP, it’s good to avoid certain things that could make your eyes worse over time.

**Foods to Stay Away From:**

1. **Sugary Foods and Drinks**
   - Too much sugar can hurt your eyes.
   - **Examples**: candy, cookies, cake, soda.
2. **Junk Food and Fast Food**
   - These often have bad fats and lots of salt.
   - **Examples**: chips, fries, burgers from fast food places.
3. **Greasy Fried Foods**
   - These can cause swelling and damage in the body.
   - **Examples**: fried chicken, onion rings.
4. **Too Much Vegetable Oil**
   - Some oils can make swelling in the body worse.
   - **Examples**: corn oil, soybean oil, sunflower oil.
5. **Too Much Alcohol**
   - Drinking a lot of alcohol can hurt your eyes over time.
   - A little is okay for some people, but not too much.
6. **High Doses of Vitamin A Without a Doctor**
   - Some people with RP take a special kind of vitamin A, but too much can hurt your liver.
   - Only take it if your eye doctor tells you to.

**Good Foods for Eye Health:**

- **Green vegetables** like spinach and kale
- **Fish** like salmon and sardines
- **Fruits** like oranges and blueberries
- **Nuts** like almonds and walnuts
- **Whole grains** like oatmeal and brown rice

Would you like a simple one-day meal plan with these foods?

What is the prognosis for retinitis pigmentosa?

Sure! Here's a simpler version:

**Retinitis pigmentosa (RP)** is an eye disease that slowly makes it harder to see over time. It affects the cells in the back of the eye (the retina), which help you see light.

**What to Expect:**

- **Starts with night blindness** – People with RP often have trouble seeing in the dark.
- **Side vision gets worse** – Over time, it’s harder to see things out of the corners of your eyes.
- **Tunnel vision** – You may feel like you’re looking through a narrow tube.
- **Later on, central vision can also get worse**, making it harder to read or recognize faces.

**How Fast It Gets Worse:**

- Some people lose their vision slowly, while others lose it faster.
- Many people with RP are considered **legally blind** by the time they are in their **40s or 50s**, but they may still see some light or shapes.

**What Affects It:**

- RP can run in families, and the way it's passed down (genetics) affects how fast it gets worse.
  - If a parent has it, the child might get a **milder form**.
  - Some forms are **more serious** and show up earlier in life.

**Can It Be Treated?**

- There is **no cure yet**, but doctors can help manage the symptoms.
- New treatments like **gene therapy** are being tested and may help some people.
- **Low vision tools**, like special glasses or magnifiers, can make daily life easier.

Would you like a short handout or infographic with this info for patients?

Can people with retinitis pigmentosa drive?

People with **retinitis pigmentosa (RP)** may be able to drive in the **early stages** of the disease, but not usually later on.

RP is an eye condition that slowly causes **vision loss**, especially **side (peripheral) vision** and **night vision**. At first, someone with RP might still have clear **central vision**, so they can read signs and see straight ahead. If their vision meets the legal standards in their state, they might be allowed to drive.

But as the disease gets worse, it becomes harder and more dangerous to drive. People with RP may:

- Have trouble seeing at night or in low light
- Miss things on the sides of the road
- Have trouble with bright lights or glare

Because of these problems, many people with RP **stop driving** as their vision changes. In the U.S., each state has its own **rules for vision and driving**, so a person with RP should get their eyes checked often and ask their eye doctor if it's still safe and legal for them to drive.

If you want, I can check what your state's rules are.

Does pregnancy have an effect on retinitis pigmentosa?

Sure! Here's a simpler explanation:

Pregnancy can sometimes cause small changes in vision for women with **retinitis pigmentosa (RP)**, but these changes are usually **temporary**.

Some women say their vision feels worse during pregnancy—they might have more trouble seeing at night or notice their side vision is a little worse. This might be because of the changes in hormones and body fluids that happen during pregnancy. But most of the time, their vision goes back to normal after the baby is born.

There’s no strong proof that pregnancy makes RP get worse forever. Doctors still recommend regular eye check-ups during pregnancy just to be safe.

Also, because RP can run in families, parents might want to talk to a genetic counselor during pregnancy to understand the chances of passing it to their children.

Let me know if you'd like a version with images or a handout-style format.

What is the likelihood that I will pass the retinitis pigmentosa gene on to my children?

Sure! Here's a simple version:

Whether you can pass retinitis pigmentosa (RP) to your children depends on the kind of gene change (mutation) you have. RP is a disease that affects eyesight, and it can be passed down in different ways:

**1. Recessive RP**

- You have two copies of the bad gene.
- Your child will usually not have RP, but they might carry the gene.
- If your partner is also a carrier, your child could have RP.

**2. Dominant RP**

- You only need one bad gene to have RP.
- Each child has a 50% chance of getting the gene and having RP.

**3. X-linked RP**

- This kind is linked to the X chromosome.
- If you’re a man with this type, your daughters may carry it, but your sons won’t get it.
- If you're a woman and carry the gene, each child has a 50% chance of getting the gene.

**What You Can Do**

To know for sure, you can get **genetic testing** and talk to a **genetic counselor**. They’ll explain your personal risk and help you plan for the future.

Would you like help finding a genetic counselor near you?

What should I do if my child is diagnosed with retinitis pigmentosa?

Sure! Here's a version written in a simple, clear way that's easy to understand:

If your child has been diagnosed with **retinitis pigmentosa (RP)**, it’s okay to feel worried. RP is an eye condition that makes it hard to see at night and over time can cause trouble seeing from the sides and sometimes in the center. Here's what you can do to help your child:

**1. Learn About the Condition**

- RP affects the eyes slowly over time.
- It can be passed down from family members.
- A **genetic counselor** can help you understand how it runs in families.

**2. Visit the Right Doctors**

- Take your child to an **eye doctor** who knows about RP.
- A **low vision specialist** can help your child use the vision they still have.
- Ask if your child can join any **research studies** or **new treatments**.

**3. Help at School**

- Talk to your child’s teacher and school staff.
- Your child may need special tools like big print books, magnifiers, or tablets.
- The school can make a plan to help your child learn in the best way.

**4. Use Tools and Make Changes at Home**

- Use **bright lights** and make things easy to see.
- Try **magnifying tools** or apps to help your child read or see better.
- Help your child stay safe—especially in the dark.

**5. Support Their Feelings**

- Your child may feel sad or different. Let them talk about their feelings.
- Show love and support.
- Find a **support group** to meet other families going through the same thing.

**6. Plan for the Future**

- Teach your child skills to be independent—like getting around safely or using special tech.
- Focus on what your child *can* do. Children with RP can still go to college, work, and do many things with the right support.

Would you like help finding a support group or ideas for school tools your child can use?

What is the extent of independence for people with advanced retinitis pigmentosa?

Sure! Here’s a version written clearly and simply for the average American adult, around a sixth-grade reading level:

**How Independent Can Someone Be with Advanced Retinitis Pigmentosa (RP)?**

People with advanced retinitis pigmentosa (RP) can still do many things on their own, but they may need some help and special tools.

### ****What Happens with Vision?****

- RP makes it hard to see at night.
- People lose side vision and may only see straight ahead (like looking through a straw).
- In late stages, central vision may also get blurry or dim.

### ****Can They Still Be Independent?****

Yes, many can still live their lives with some help:

#### **Getting Around**

- They can walk using a white cane or with a guide dog.
- They learn how to travel safely with training.
- Some use GPS apps that speak directions.

#### **Reading and Using Technology**

- Special tools can read text out loud.
- They can use phones and computers with voice commands.
- Books and websites can be read with audio or large print.

#### **Work**

- Many people with RP still work.
- Jobs that use talking, typing, or listening can work well.
- Workplaces may provide helpful tools or changes.

#### **At Home**

- People use voice-controlled lights or appliances.
- Tactile labels and smart devices help with cooking, cleaning, and more.

### ****What Helps Most?****

- Vision rehab programs
- Technology like screen readers
- Support from family or groups
- Learning new ways to do things

### ****What’s Hard?****

- Moving safely in new places
- Seeing at night or in bright sunlight
- Feeling lonely or sad if they lose more vision

### ****Bottom Line****

People with advanced RP can still live full, active lives. They just do things a little differently—with the right tools, training, and support.

Would you like this turned into a printable handout or slide?

What part of the eye is affected by retinitis pigmentosa?

Retinitis pigmentosa is an eye disease that affects the **retina**, which is the thin layer of tissue at the back of your eye. The retina helps you see by sending images to your brain.

In this disease, special cells in the retina called **rods and cones** slowly stop working. Rods help you see in the dark, and cones help you see details and colors. When these cells stop working, people may have trouble seeing at night or lose side vision. Over time, it can become hard to see clearly, even in daylight.

Let me know if you'd like a simple drawing or explanation for kids, too!

Can women get retinitis pigmentosa?

Yes, women can get retinitis pigmentosa (RP).

RP is an eye disease that people are usually born with. It slowly causes vision loss over time. Both men and women can have it.

In some families, the disease is passed down from parents to children. Sometimes, women may not have as many symptoms as men, but they can still be affected.

If someone in your family has RP, it’s a good idea to talk to an eye doctor or a genetic counselor to learn more.

How long is the average lifespan of a person with retinitis pigmentosa?

People with retinitis pigmentosa (RP) usually live a normal life. RP affects the eyes, not the rest of the body. It makes it hard to see at night and causes vision to slowly get worse over time. Some people may lose most of their sight, but it doesn't usually shorten how long they live.

In some rare cases, RP is part of a bigger health problem, like Usher or Bardet-Biedl syndrome. These can affect other parts of the body and may cause more health issues. But most people with RP live just as long as anyone else.

Is retinitis pigmentosa a disability?

Yes, **retinitis pigmentosa (RP)** is considered a **disability**.

RP is an eye disease that makes it harder to see over time. People with RP may have trouble seeing at night or lose their side (peripheral) vision. In some cases, they may go blind.

Because it can make daily life difficult—like reading, walking safely, or driving—RP is seen as a disability.

People with RP may get help such as:

- Special tools to help them see better
- Extra support at school or work
- Disability benefits or services

Even though there’s no cure yet, many people with RP learn ways to live full and active lives.

What does a person with retinitis pigmentosa see?

Sure! Here's a simple explanation:

**What does a person with retinitis pigmentosa see?**

Retinitis pigmentosa (RP) is an eye disease that slowly makes it harder to see. It affects the light-sensing cells in the back of the eye.

At first, people with RP often have trouble seeing at night or in the dark. This is called **night blindness**. They might bump into things in dim places, like movie theaters.

As time goes on, they start to lose **side vision**. It feels like they are looking through a tunnel. This is called **tunnel vision**. They can still see straight ahead, but not to the sides.

Later, they may also lose their **central vision**, which makes it hard to read, see faces, or do close-up work. Some people with RP may become legally blind.

They may also see **flashing lights** or have trouble with **bright light**.

Would you like me to show an image to help explain what this looks like?

How quickly does retinitis pigmentosa progress?

Retinitis pigmentosa (RP) is an eye disease that gets worse slowly over time. It affects how well you can see, especially at night and from the sides.

**Here's how it usually goes:**

- **Early signs** often start in **childhood or the teen years**.
- The first problem is usually **trouble seeing at night**.
- Over time, **side vision (peripheral vision)** gets smaller—like looking through a tunnel.
- **Reading and seeing straight ahead (central vision)** usually stays okay until later in life, often into the **40s or 50s**.
- Some people lose most of their vision by middle age, but others keep some vision for much longer.

**How fast it gets worse depends on:**

- **Your genes (family history)**.
- **The type of RP you have**—some kinds move faster than others.
- **Other health problems**, like hearing loss (in Usher syndrome).

There is no cure yet, but doctors are working on new treatments like gene therapy and special devices to help people see better.

Let me know if you’d like a simple chart or image to explain this better!

What if a person suffering from retinitis pigmentosa develops cataract?

Sure! Here's a simplified version:

If someone has **retinitis pigmentosa (RP)** and then gets a **cataract**, their vision can get even worse. RP is a disease that slowly damages the eyes and makes it hard to see, especially at night or from the sides. A cataract makes the eye’s lens cloudy, like a foggy window, and makes things look blurry.

The good news is that **cataract surgery** can help. Doctors can remove the cloudy lens and put in a clear one. This often makes **central vision** (the part you use to read and see faces) a little better. But the vision problems from RP, like poor side vision or night vision, don’t go away.

Before surgery, the doctor will check the eyes carefully to make sure the surgery will help. Most people with RP who get cataract surgery say they can see better afterward and feel happier with their vision.

Let me know if you want a version with visuals or for patient education!

Does "night-blindness" mean a person has retinitis pigmentosa?

No, night-blindness does not always mean a person has retinitis pigmentosa (RP).

**Night-blindness** means someone has trouble seeing in the dark or in low light. This can happen for many reasons. One reason might be **retinitis pigmentosa**, which is a rare eye disease that gets worse over time. But night-blindness can also be caused by other things, like:

- Not getting enough **vitamin A** in your diet
- Having **cataracts**, which make the lens of the eye cloudy
- Being very **nearsighted**
- Having certain **inherited conditions** that don’t get worse

If someone has night-blindness, they should see an eye doctor to find out the cause. It's a symptom, not a diagnosis by itself.

Which doctor should you consult for retinitis pigmentosa?

If you have **retinitis pigmentosa**, you should see an **eye doctor called a retina specialist**. This doctor is trained to look at the back part of your eye, called the retina, and can help check how the disease is affecting your vision.

You might also visit a **low vision specialist**. This is another kind of eye doctor who helps people use their remaining sight as best as possible. They can show you special glasses or tools to help you see better.

Because retinitis pigmentosa can run in families, a **genetic counselor** may also be helpful. They talk to you about how the condition is passed on and if others in your family might be at risk.

If you want, I can help you find a retina specialist near you.
