# Supplementary material for: Evaluation of the artificial intelligence chatbots in frequently asked questions about retinitis pigmentosa: a comparative analysis between ChatGPT-4 and Gemini-2.0
Source: Int J Retina Vitreous. 2025 Nov 28;12:1. doi: 10.1186/s40942-025-00772-4 (PMC12763912; doi:10.1186/s40942-025-00772-4)
Supplement: Supplementary file 3 — Supplementary Material 3 [file 40942_2025_772_MOESM3_ESM.docx]

What is Retinitis Pigmentosa?

Retinitis pigmentosa (RP) is a group of inherited eye diseases that cause progressive vision loss. Here's a breakdown of key aspects:

- **Nature of the Disease:**
  - It's a genetic disorder affecting the retina, the light-sensitive tissue at the back of the eye.
  - It involves the gradual breakdown and loss of photoreceptor cells (rods and cones) in the retina.
- **Symptoms:**
  - The most common early symptom is difficulty seeing in low light or at night (night blindness).
  - As the disease progresses, it typically leads to a gradual loss of peripheral (side) vision, resulting in "tunnel vision."
  - In later stages, central vision can also be affected.
  - Other symptoms can include:
    - Problems with color vision.
    - Sensitivity to bright light (photophobia).
- **Causes:**
  - RP is caused by genetic mutations.
  - These mutations disrupt the function of the retinal cells.
  - It can be inherited in various patterns (autosomal recessive, autosomal dominant, or X-linked).
- **Progression and Impact:**
  - The rate of vision loss varies significantly among individuals.
  - While some people experience slow progression, others may have more rapid vision decline.
  - It can lead to significant visual impairment, and in some cases, blindness.
- **Important considerations:**
  - There is currently no cure for RP.
  - Management focuses on slowing progression, and helping people maintain the vision they have.
  - Genetic counseling is important for those with a family history of RP.

I hope this helps.

What causes you to get Retinitis Pigmentosa?

Retinitis pigmentosa (RP) is primarily caused by genetic mutations. Here's a breakdown:

- **Genetic Mutations:**
  - RP is a group of inherited eye disorders. This means it's usually passed down through families.
  - The condition arises from mutations in various genes that affect the retina's light-sensitive cells (photoreceptors).
  - There are many different genes associated with RP, and mutations in any of them can lead to the disease.
  - These genetic mutations disrupt the normal function of the retinal cells, causing them to gradually deteriorate.
- **Inheritance Patterns:**
  - RP can be inherited in different ways:
    - **Autosomal dominant:** Only one copy of the mutated gene is needed to develop the condition.
    - **Autosomal recessive:** Two copies of the mutated gene (one from each parent) are required.
    - **X-linked:** The mutated gene is located on the X chromosome, which affects males more severely.

- **Sporadic Cases:**
  - While most RP cases are inherited, some occur sporadically, meaning they arise from new genetic mutations that are not inherited from parents.
  - This means that there are cases of retinitis pigmentosa where no family history of the disease is present.
- In summary, Retinitis Pigmentosa is a condition mostly caused by gene mutations.

Can Retinitis Pigmentosa be passed down through heredity?

Yes, Retinitis Pigmentosa (RP) is indeed an inherited disorder. This means it can be passed down through heredity. Here's a breakdown of how it's inherited:

- **Genetic Basis:**
  - RP is caused by genetic mutations. These mutations affect the cells in the retina, leading to progressive vision loss.
- **Inheritance Patterns:**
  - RP can be inherited in several ways:
    - **Autosomal recessive:**
      - This is the most common pattern.
      - It requires inheriting two copies of the mutated gene, one from each parent.
      - Parents may be carriers but not show symptoms.
    - **Autosomal dominant:**
      - Only one copy of the mutated gene is needed to develop the condition.
      - One parent typically has RP.
    - **X-linked:**
      - The mutated gene is located on the X chromosome.
      - This pattern affects males more severely.
      - Females can be carriers or also be affected.

- **Genetic Complexity:**
  - There are many different genes associated with RP, which contributes to the variability of the disease.

In summary, genetic factors play a significant role in RP, and understanding the inheritance patterns is crucial for genetic counseling and family planning.

Can Retinitis Pigmentosa be caused by injury or accident?

Based on the information available, Retinitis Pigmentosa (RP) is primarily a genetic disorder. This means it's usually caused by inherited gene mutations. However, here's a breakdown:

- **Genetic Basis:**
  - The vast majority of RP cases are due to genetic factors. These gene mutations affect the retina's photoreceptor cells.
  - It's an inherited disease.
- **Injury and Other Factors:**
  - While very rare, some sources do state that in rare cases eye injury could be a cause.
  - It is much more common that RP is a result of genetics.
  - So while the overwhelming number of cases are genetic, it is possible that in some rare instances, there could be a link to eye injury.

In summary, while the primary cause of RP is genetic, it's important to acknowledge that in very rare circumstances, other factors, including eye injuries, might play a role.

What are the symptoms of Retinitis Pigmentosa?

Retinitis pigmentosa (RP) encompasses a group of inherited eye diseases that progressively damage the retina. This damage leads to a gradual decline in vision. Here's a breakdown of the common symptoms:

- **Night blindness (nyctalopia):**
  - This is often one of the earliest symptoms. Individuals may find it increasingly difficult to see in dim light or at night.
  - Adapting from bright to dark environments becomes challenging.

- **Loss of peripheral vision:**
  - This results in a narrowing of the field of vision, often described as "tunnel vision."
  - Individuals may bump into objects at their sides because they cannot see them.
- **Difficulty with central vision:**
  - In later stages, central vision can also be affected, leading to blurred vision and difficulty with tasks requiring sharp vision, such as reading.
- **Problems with color vision:**
  - Some individuals with RP may experience a decline in their ability to distinguish colors.
- **Sensitivity to light (photophobia):**
  - Some individuals can develop a increased sensitivity to bright lights.

It's important to note that:

- The progression of RP varies significantly among individuals.
- Symptoms may begin in childhood or early adulthood.
- While RP can lead to significant vision loss, not everyone with the condition becomes completely blind.

If you are experiencing any of these symptoms, it is very important to see an eye care professional.

How can Retinitis Pigmentosa be diagnosed?

Diagnosing Retinitis Pigmentosa (RP) involves a combination of clinical evaluation and specialized tests. Here's a breakdown of the typical diagnostic process:

**1. Comprehensive Eye Examination:**

- **Ophthalmoscopy:**
  - This involves examining the retina with a specialized instrument. In RP, characteristic changes like black bone-spicule pigment deposits may be visible.
- **Visual Acuity Test:**
  - This standard eye chart test assesses how well you can see.
- **Visual Field Testing:**
  - This test measures your peripheral (side) vision, which is often affected early in RP.
- **Color Vision Testing:**
  - This evaluates your ability to distinguish colors, which can also be impaired in RP.

**2. Specialized Tests:**

- **Electroretinography (ERG):**
  - This is a crucial test that measures the electrical activity of the retina in response to light. It helps determine the extent of retinal cell dysfunction.
- **Optical Coherence Tomography (OCT):**
  - This imaging technique provides detailed cross-sectional images of the retina, allowing doctors to assess its structure and identify abnormalities.
- **Genetic Testing:**
  - Because RP is a genetic disorder, genetic testing can identify specific gene mutations associated with the condition. This can be very helpful for confirming the diagnosis, determining the inheritance pattern, and sometimes providing prognostic information.

**3. Medical History and Family History:**

- A thorough medical history, including any family history of vision problems, is essential.

In summary, the diagnosis of RP relies on a combination of:

- Clinical eye examination.
- Functional testing (ERG, visual field testing).
- Imaging (OCT).
- Genetic testing.

I hope this information is helpful.

How does Retinitis Pigmentosa affect vision?

Retinitis pigmentosa (RP) is a group of inherited eye diseases that progressively damage the retina, the light-sensitive tissue at the back of the eye. This damage leads to a gradual decline in vision, and it affects vision in several key ways:

- **Night Blindness:**
  - This is often the first symptom. RP primarily affects rod cells, which are responsible for vision in low-light conditions. As these cells deteriorate, individuals experience increasing difficulty seeing at night or in dimly lit environments.
- **Peripheral Vision Loss (Tunnel Vision):**
  - As the disease progresses, the field of vision narrows, resulting in "tunnel vision." This means that while central vision may remain relatively intact for some time, the ability to see things to the sides diminishes.
- **Central Vision Loss:**
  - In later stages, RP can also affect cone cells, which are responsible for central vision and color vision. This can lead to decreased visual acuity, making it difficult to see fine details or read.
- **Color Vision Problems:**
  - Damage to cone cells can also result in difficulty distinguishing colors.
- **Sensitivity to Light (Photophobia):**
  - some people with RP experience increased sensitivity to bright lights.

In summary, RP causes a progressive loss of vision, typically starting with night blindness and peripheral vision loss, and potentially leading to central vision loss and color vision problems.

Can Retinitis Pigmentosa cause complete blindness?

Yes, Retinitis Pigmentosa (RP) can indeed lead to complete blindness, although the progression of the disease varies significantly from person to person. Here's a breakdown:

- **Progressive Vision Loss:**
  - RP is a group of inherited retinal diseases that cause a gradual deterioration of the light-sensitive cells in the retina.
  - This typically begins with night blindness and a loss of peripheral vision, leading to "tunnel vision."
  - Over time, the central vision can also be affected.
- **Potential for Complete Blindness:**
  - While not everyone with RP will experience total blindness, it is a possible outcome, especially in advanced stages of the disease.
  - The rate of vision loss varies greatly, and some individuals may retain some degree of useful vision throughout their lives.
- **Key points:**
  - The disease causes damage to the photoreceptor cells in the eyes over time, often resulting in progressive vision loss.
  - It is important to understand that even if complete blindness does not occur, that many people with RP do become legally blind.

In summary, while the progression of RP is different for everyone, it is a disease that can lead to significant vision loss, including complete blindness.

Can we stop Retinitis Pigmentosa from progressing?

Unfortunately, there is currently no cure for retinitis pigmentosa (RP), and therefore, we cannot definitively "stop" its progression. However, there are strategies and treatments aimed at slowing its progression and managing its symptoms. Here's a breakdown:

**Key Points:**

- **No Cure, but Management:**
  - While a complete halt to RP's progression isn't yet possible, medical advancements focus on slowing it down and preserving existing vision.
- **Management Strategies:**
  - **Regular Eye Exams:** Crucial for monitoring progression and addressing complications.
  - **Vitamin A and Omega-3 Fatty Acids:** Some studies suggest these may help slow progression in certain individuals.
  - **Protecting from Sunlight:** Wearing UV-protective sunglasses is essential.
  - **Low Vision Aids:** Devices and technologies can help maximize remaining vision.
  - **Gene Therapy:**
    - For a specific type of RP caused by mutations in the RP65 gene, gene therapy (Luxturna®) is an approved treatment.
  - **Treating Associated Conditions:** Managing conditions like cataracts or cystoid macular edema can improve vision.
- **Ongoing Research:**
  - Research into gene therapies and other potential treatments is ongoing, offering hope for future advancements.

**In summary:**

While RP cannot be stopped entirely, proactive management and ongoing research offer ways to slow its progression and improve quality of life. It is very important that those with RP maintain close contact with their ophthalmologist.

Can Retinitis Pigmentosa be reversed?

Based on the information available, here's a breakdown regarding the reversibility of Retinitis Pigmentosa (RP):

- **Current State of Treatments:**
  - Currently, there is no broad cure that can fully reverse the damage caused by RP.
  - However, there are treatments available that can help slow the progression of the disease, and in some specific genetic cases, there are gene therapies that can help.
  - Specifically there is an FDA approved gene therapy for RPE65 gene related RP, named Luxturna.
- **Focus on Slowing Progression:**
  - Much of the current medical approach focuses on managing the disease and slowing its progression to preserve existing vision for as long as possible.
  - This includes:
    - Vitamin and nutritional supplements.
    - Visual aids and rehabilitation.
    - Photo-protection.
- **Emerging Therapies:**
  - Research into gene therapies and other innovative treatments, such as CRISPR technology, is ongoing and showing promise.
  - These therapies aim to target the underlying genetic causes of RP and potentially restore lost vision.
  - There are new gene therapies, that are in the development stages.
- **Key takeaway:**
  - While RP is not generally considered reversible, advancements in genetic therapies are providing hope for future treatments that may be able to slow, stop, or even reverse some of the vision loss.

It's important to consult with an ophthalmologist or retina specialist for accurate and personalized information about RP and the available treatment options.

Can sunlight exposure worsen vision of people with Retinitis Pigmentosa?

Yes, sunlight exposure can indeed worsen the vision of people with Retinitis Pigmentosa (RP). Here's why:

- **Increased Sensitivity:**
  - People with RP often experience photophobia, which is an increased sensitivity to light. This means bright light, including sunlight, can cause discomfort and pain.
  - The progressive damage to the retinal cells in RP makes the eyes more vulnerable to the harmful effects of ultraviolet (UV) radiation.
- **UV Damage:**
  - UV rays, particularly UV-A, can damage the macula, a central part of the retina. This damage can accelerate the deterioration of retinal cells, which is already happening in people with RP.
  - Therefore, exposure to sun light can increase the rate of damage to the retina.
- **Glare:**
  - People with RP often experience increased glare, which can make it very difficult to see in bright light conditions.

Therefore, it is very important for people with RP to take precautions to protect their eyes from sunlight. This includes:

- Wearing sunglasses with 100% UV protection.
- Wearing a wide-brimmed hat.
- Avoiding prolonged exposure to bright sunlight.

I hope this information is helpful.

Can I treat Retinitis Pigmentosa by nutritional care?

While nutritional care cannot cure Retinitis Pigmentosa (RP), it can play a supportive role in maintaining overall eye health and potentially slowing down the progression of the disease. Here's a breakdown of what that entails:

**Key Nutritional Considerations:**

- **Vitamin A:**
  - Some studies have explored the role of Vitamin A in RP. However, it's crucial to understand that high-dose Vitamin A supplementation can be harmful. Therefore, it is important to only take Vitamin A supplements under the guidance of a medical professional.
  - It is very important to have your Vitamin A levels monitored by your doctor.
- **Omega-3 Fatty Acids:**
  - These fatty acids, found in fish oil, may help support retinal health.
- **Lutein and Zeaxanthin:**
  - These antioxidants, present in leafy green vegetables, can protect the eyes from oxidative damage.
- **A Balanced Diet:**
  - A diet rich in fruits, vegetables, and whole grains provides essential nutrients for overall health, including eye health.
- **Vitamin E:**
  - It is important to know that some studies have shown that Vitamin E can have a damaging effect on retinitis pigmentosa. Therefore it is recommended that people with RP avoid high doses of Vitamin E supplements.

**Important Considerations:**

- **Consult a Healthcare Professional:** It's crucial to consult with an ophthalmologist or a registered dietitian before making significant dietary changes or taking supplements. They can provide personalized recommendations based on your individual needs.
- **No Cure:** It's essential to understand that nutritional care is not a cure for RP. It's a supportive measure to maintain overall health and potentially slow down disease progression.
- **Lifestyle Factors:** In addition to diet, other lifestyle factors, such as avoiding smoking and protecting your eyes from excessive sunlight, are also important for eye health.

In summary, while a healthy diet can support eye health, it's not a cure for RP. Always consult with a healthcare professional for personalized guidance.

Is Retinitis Pigmentosa treatable?

It's important to understand that while there isn't a "cure" for retinitis pigmentosa (RP) in the sense of reversing the damage already done, there are treatments and management strategies that can help slow its progression and improve quality of life. Here's a breakdown:

- **Current State of Treatment:**
  - Currently, there is no cure for most forms of retinitis pigmentosa.
  - However, research is ongoing, and there are some treatments that can help manage the condition.
  - One FDA-approved gene therapy, voretigene neparvovec (Luxturna), exists, but it's only for a very specific and rare form of RP caused by mutations in the RPE65 gene.
- **Management and Potential Treatments:**
  - **Vitamin A and DHA:** Some studies suggest that vitamin A palmitate and docosahexaenoic acid (DHA) may slow the progression of RP. However, it's crucial to take these under the guidance of a doctor.
  - **Gene Therapy:** As mentioned, Luxturna is available for a specific genetic cause of RP. Research into gene therapy for other genetic mutations is ongoing.
  - **Low-Vision Aids:** Devices and strategies can help people with RP maximize their remaining vision.
  - **Treating Complications:** Conditions like cataracts and macular edema, which can accompany RP, can often be treated.
  - **Ongoing Research:** There is much research being done on things like:
    - Artificial retinal implants.
    - Other forms of gene therapy.
    - Stem cell therapy.
- **Key Takeaways:**
  - While a general cure remains elusive, there are ways to manage RP and slow its progression.
  - It is very important to consult with an ophthalmologist that specializes in retinal diseases.
  - Research is ongoing and new treatments are being developed.

I hope this information is helpful.

What should I eat if I have Retinitis Pigmentosa?

It's important to understand that while diet can support overall eye health, it's not a cure for Retinitis Pigmentosa (RP). RP is a genetic disorder, and its progression varies. However, certain nutrients are believed to be beneficial for eye health and may help support the remaining retinal function. Here's a breakdown of dietary recommendations:

**Foods to Include:**

- **Foods Rich in Antioxidants:**
  - **Green leafy vegetables:** Spinach, kale, broccoli, and collard greens are excellent sources of lutein and zeaxanthin, which protect the eyes from free radical damage.
  - **Brightly colored fruits:** Berries, oranges, and other colorful fruits are rich in vitamins A, C, and E, which are important antioxidants.
- **Omega-3 Fatty Acids:**
  - Fatty fish like salmon, mackerel, and tuna are excellent sources of omega-3s, which are essential for retinal health.
- **Vitamin A:**
  - Dairy products (milk, eggs, cheese), and liver are good sources of Vitamin A. However, it is important to note that very high dosages of vitamin A can be toxic, so it is important to speak with your doctor before taking vitamin A supplements.
- **Zinc:**
  - Nuts, seeds, and whole grains contain zinc, which plays a role in maintaining healthy vision.

**Key Nutrients and Why They're Important:**

- **Lutein and Zeaxanthin:** These antioxidants are concentrated in the macula, the part of the retina responsible for central vision.
- **Omega-3 Fatty Acids:** These healthy fats support the structure and function of the retina.
- **Vitamin A:** Essential for night vision and overall retinal health.
- **Zinc:** Helps transport vitamin A to the retina.

**Important Considerations:**

- **Consult with a healthcare professional:** It is very important that you talk to your ophthalmologist or a registered dietitian. They can provide personalized dietary advice based on your specific condition and health needs.
- **Supplementation:** While a healthy diet is crucial, some individuals with RP may benefit from specific vitamin or mineral supplements. Again, consult with your doctor before starting any supplements.
- **Balanced Diet:** Focus on a well-rounded diet that includes a variety of fruits, vegetables, whole grains, and lean proteins.
- **Avoid excessive sugar:** High sugar consumption can contribute to various health problems, including those affecting eye health.

By focusing on a nutrient-rich diet and working closely with your healthcare providers, you can take proactive steps to support your eye health.

What foods should I avoid if I have Retinitis Pigmentosa?

While there's no specific "Retinitis Pigmentosa diet" that will cure the condition, dietary choices can play a role in overall health, including eye health. Here's a breakdown of foods that are generally recommended to limit or avoid, based on information found:

- **Saturated and Trans Fats:**
  - These fats, found in foods like:
    - Cheese
    - Sausages
    - Butter
    - Processed snacks and baked goods
  - Can contribute to circulatory problems, which may negatively impact blood flow to the retina.
- **Fried Foods:**
  - These foods can increase bad cholesterol and blood pressure, which are detrimental to overall health and can affect eye health.
- **Sugary Drinks:**
  - High sugar intake can increase the risk of:
    - Type 2 diabetes
    - Heart disease
    - Which can indirectly affect eye health.

- **Refined White Foods:**
  - Foods like white bread, white rice, and pasta offer little nutritional value and can contribute to blood sugar spikes. It is better to opt for whole grain alternatives.

Key considerations:

- Maintaining a healthy weight and cardiovascular health is important for overall eye health.
- A balanced diet rich in antioxidants, vitamins, and minerals is generally recommended.
- It is always best to consult with your eye doctor and a registered dietitian for personalized dietary advice.

I hope this helps.

What is the prognosis for retinitis pigmentosa?

Retinitis pigmentosa (RP) is a group of inherited disorders that progressively damage the retina. Therefore, the prognosis involves a gradual decline in vision. Here's a breakdown of key aspects:

- **Progressive Vision Loss:**
  - RP typically begins with night blindness (difficulty seeing in low light) and a gradual loss of peripheral vision.
  - Over time, this can lead to "tunnel vision," where only central vision remains.
  - In advanced stages, central vision can also be affected, leading to significant visual impairment.
  - The rate of progression varies significantly between individuals.
- **No Cure, but Management:**
  - Currently, there's no cure for RP.
  - However, research is ongoing, and there are treatments to manage certain aspects of the disease and slow its progression in certain circumstances.
  - For a small subset of patients with RP caused by mutations in the RPE65 gene, gene therapy (Luxturna) is available.
  - Management also includes:
    - Regular eye exams
    - Low vision aids
    - Protecting the eyes from UV light
    - Management of complications like cataracts and macular edema.
- **Variability:**
  - The onset and rate of progression of RP vary significantly.
  - Genetic testing can help determine the specific type of RP, which may provide some insight into the potential course of the disease.
- **Ongoing Research:**
  - There is continuing research being done, into potential treatments, including other forms of gene therapy.

It's crucial for individuals with RP to work closely with an ophthalmologist to monitor their condition and explore available management options.

Can people with retinitis pigmentosa drive?

Driving with retinitis pigmentosa (RP) is complex and depends heavily on the individual's stage of the disease. Here's a breakdown of the key factors:

- **Progressive Vision Loss:**
  - RP causes progressive damage to the retina, leading to night blindness, loss of peripheral vision (tunnel vision), and eventually, reduced central vision.
  - This progressive nature means that a person's ability to drive can change over time.
- **Impact on Driving:**
  - Night blindness makes driving in low-light conditions extremely hazardous.
  - Loss of peripheral vision significantly impairs a driver's ability to see hazards coming from the sides.
  - As the disease advances, even daytime driving can become unsafe.
- **Legal and Safety Considerations:**
  - Driving regulations vary by state or region, with specific vision requirements that drivers must meet.
  - Individuals with RP should regularly have their vision checked and comply with these regulations.
  - Safety is paramount. Even if legally allowed to drive, individuals must assess their own ability to do so safely.
- **Adaptations and Alternatives:**
  - In early stages, some individuals may use low-vision aids to extend their driving ability.
  - However, as RP progresses, alternative transportation options become essential.

In summary, while some people with early-stage RP may be able to drive, the progressive nature of the disease often leads to driving limitations and eventually necessitates alternative transportation. It is very important that those with RP have regular eye exams, and adhere to driving laws.

Does pregnancy have an effect on retinitis pigmentosa?

Based on the information available, pregnancy can indeed have an effect on retinitis pigmentosa (RP). Here's a summary of key findings:

- **Potential for Worsening Visual Function:**
  - Studies indicate that pregnancy may lead to a worsening of visual acuity and a decrease in the visual field in women with RP.
  - Specifically, research has shown that women who have given birth appear to have a higher risk of experiencing significant central vision loss compared to those who have not.
- **Factors and Observations:**
  - Research has shown that women who have had children, had a higher risk of reaching a visual acuity below a certain level.
  - It is being studied if the hormonal changes during pregnancy, could be a factor in the worsening of the RP.
  - There are studies that focus on the USH2A gene, and how that gene effects the progression of RP during pregnancies.
- **Importance of Further Research:**
  - While these findings suggest a link between pregnancy and RP progression, further research is necessary to fully understand the underlying mechanisms.
  - These findings are important for family planning for women that have RP.

In essence, it's crucial for women with RP to discuss potential risks and considerations with their ophthalmologist and obstetrician before and during pregnancy.

What is the likelihood that I will pass the retinitis pigmentosa gene on to my children?

The likelihood of passing on the retinitis pigmentosa (RP) gene to your children depends heavily on the specific genetic inheritance pattern involved. RP can be inherited in several ways:

- **Autosomal Dominant:**
  - If you have autosomal dominant RP, there's a 50% chance that each of your children will inherit the gene and potentially develop the condition.
- **Autosomal Recessive:**
  - If you have autosomal recessive RP, it means you inherited a copy of the mutated gene from each of your parents. In this case:
    - If your partner is not a carrier of the RP gene, your children will be carriers but likely won't develop the condition.
    - If your partner is also a carrier, there's a 25% chance your child will have RP, a 50% chance they'll be a carrier, and a 25% chance they won't inherit the gene.
- **X-linked:**
  - This inheritance pattern is related to the X chromosome. The likelihood of passing on the gene varies depending on whether you are male or female:
    - If a mother carries an X-linked RP gene, there's a 50% chance her sons will have RP and a 50% chance her daughters will be carriers.
    - Fathers that have the X-linked RP, will pass the gene to all of their daughters, making them carriers, but will not pass the gene to their sons.

- **Sporadic Cases:**
  - In some cases, RP occurs with no known family history. This is referred to as a sporadic case. The risk of passing this form of RP on to your children is significantly lower, but still possible due to the fact that new mutations can occur.

**Key Points:**

- Genetic testing is crucial to determine the specific inheritance pattern of RP in your family.
- Genetic counseling can provide personalized information about your risk of passing on the gene and help you make informed decisions.

It is very important to consult with a medical professional, and especially a genetic counselor, for accurate and personalized information.

What should I do if my child is diagnosed with retinitis pigmentosa?

Receiving a diagnosis of retinitis pigmentosa (RP) for your child can be overwhelming. Here's a breakdown of important steps and considerations:

**1. Seek Expert Medical Care:**

- **Ophthalmologist specializing in retinal diseases:**
  - It's crucial to find an ophthalmologist with expertise in RP. They can provide accurate diagnosis, monitor progression, and recommend appropriate management strategies.
- **Genetic counseling:**
  - RP is a genetic condition. Genetic counseling can help you understand the inheritance pattern, assess the risk for other family members, and explore potential genetic testing options.
- **Regular eye exams:**
  - Consistent monitoring of your child's vision is essential to track the progression of RP and address any related eye problems.

**2. Understanding the Condition:**

- **Educate yourself:**
  - Learn as much as possible about RP, its symptoms, and its progression. Reliable sources like the Foundation Fighting Blindness and reputable medical websites can provide valuable information.
- **Symptoms to watch for:**
  - Be aware of common symptoms, including:
    - Night blindness (difficulty seeing in low light)
    - Loss of peripheral vision (tunnel vision)
    - Difficulty with color vision
    - Sensitivity to bright light

**3. Supporting Your Child:**

- **Early intervention:**
  - If your child experiences vision loss, early intervention services can help them develop adaptive skills and maximize their remaining vision.
- **Educational support:**
  - Work with your child's school to ensure they receive appropriate accommodations, such as preferential seating, assistive technology, and modifications to assignments.
- **Emotional support:**
  - RP can have a significant emotional impact on your child and your family. Seek support from counselors, therapists, or support groups to help cope with the challenges of the condition.
- **Promote independence:**
  - Encourage your child to participate in activities and develop independence, while providing necessary support and adaptations.

**4. Exploring Potential Treatments and Research:**

- **Stay informed about research:**
  - Research on RP is ongoing, and new treatments are being developed. Stay informed about clinical trials and potential therapies.
- **Assistive devices:**
  - Explore assistive devices, such as low-vision aids, to help your child maximize their remaining vision.
- **Healthy lifestyle:**
  - A healthy lifestyle, including a balanced diet and regular exercise, can support overall well-being.

**Key considerations:**

- **Genetic testing:** Genetic testing can help to find out the specific gene mutation causing the RP, which is becoming increasingly important, as some gene therapies are gene specific.
- **Support organizations:** Groups like The Foundation Fighting Blindness can provide very valuable support and information.

It is very important to maintain close contact with you and your childs opthamologist, as the information regarding retinitis pigmentosa is consistently improving.

What is the extent of independence for people with advanced retinitis pigmentosa?

The extent of independence for people with advanced retinitis pigmentosa (RP) varies significantly, but it's important to understand that even with advanced RP, many individuals maintain a degree of independence. Here's a breakdown of factors influencing independence and available support:

**Challenges to Independence:**

- **Progressive Vision Loss:**
  - RP causes a gradual loss of photoreceptor cells in the retina, leading to night blindness, loss of peripheral vision (tunnel vision), and eventually, central vision loss.
  - In advanced stages, this can severely limit mobility, reading, and the ability to perform daily tasks.
- **Impact on Daily Activities:**
  - Activities like driving, navigating unfamiliar environments, and even simple household chores become increasingly difficult.
  - Social interactions can also be affected due to difficulty recognizing faces and interpreting visual cues.

**Factors Supporting Independence:**

- **Adaptive Strategies and Technologies:**
  - **Low Vision Aids:** Magnifiers, telescopes, and electronic devices can help maximize remaining vision.
  - **Assistive Technology:** Screen readers, voice-activated devices, and specialized software enable access to computers and smartphones.
  - **Orientation and Mobility Training:** Learning to use canes, guide dogs, and other techniques allows for safe navigation.
  - **Home Modifications:** Improved lighting, organized spaces, and safety measures (handrails, etc.) enhance safety and ease of movement.
- **Support Systems:**
  - **Rehabilitation Services:** Occupational therapists and mobility specialists provide training and support.
  - **Support Groups:** Connecting with others with RP offers emotional support and practical advice.
  - **Social Services:** Local agencies can provide information on resources and financial assistance.

**Key Considerations:**

- While advanced RP presents significant challenges, individuals can maintain a level of independence with appropriate support and adaptations.
- The degree of independence is influenced by the individual's rate of disease progression, access to resources, and personal coping strategies.
- It is very important to get help from professionals that specialize in low vision, and blindness.

In summary, although advanced retinitis pigmentosa does present significant challenges to independence, there are many tools and support structures that can help people maintain a high quality of life.

What part of the eye is affected by retinitis pigmentosa?

Retinitis pigmentosa (RP) primarily affects the:

- **Retina:**
  - Specifically, the photoreceptor cells within the retina, which are the rod and cone cells.
  - The retinal pigment epithelium (RPE) is also affected.

Here's a breakdown:

- **Rod cells:**
  - These are responsible for night vision and peripheral vision.
  - RP often initially affects rod cells, leading to night blindness and loss of peripheral vision (tunnel vision).
- **Cone cells:**
  - These are responsible for central vision and color vision.
  - As RP progresses, cone cells can also be affected, leading to loss of central vision and color vision difficulties.

In essence, RP is a degenerative disease that progressively damages the light-sensitive cells in the retina.

Can women get retinitis pigmentosa?

Yes, women can get retinitis pigmentosa (RP). However, the way it manifests can differ due to genetic inheritance patterns, particularly with X-linked RP. Here's a breakdown:

- **General RP:**
  - RP is a group of inherited disorders that affect the retina. It can be inherited in several ways, including autosomal recessive, autosomal dominant, and X-linked.
  - So, regardless of gender anyone whom inherits the genes that cause RP can get the condition.
- **X-linked RP:**
  - This inheritance pattern involves genes on the X chromosome.
  - Because women have two X chromosomes, they can be carriers of the X-linked RP gene.
  - While some female carriers may show no symptoms or have mild symptoms, others can experience significant vision loss.
  - Men, having only one X chromosome, are more likely to exhibit more severe symptoms of X-linked RP if they inherit the affected gene.
- **Key points:**
  - Women can inherit and express RP.
  - The severity of RP in women can vary.
  - Genetic counseling is very important when there is a family history of retinitis pigmentosa.

How long is the average lifespan of a person with retinitis pigmentosa?

Based on the information available, retinitis pigmentosa (RP) itself does not typically affect a person's lifespan. Here's a breakdown:

- **Normal Life Expectancy:**
  - In most cases, when RP affects only the eyes (nonsyndromic RP), individuals have a normal life expectancy.
- **Factors to Consider:**
  - It's important to note that RP is a degenerative eye disease that primarily impacts vision.
  - The severity and progression of vision loss can vary significantly among individuals.
  - It is also importatnt to note that in some rare cases RP is associated with other syndromic conditions, and those conditions may have an affect on lifespan.

In summary, having retinitis pigmentosa does not, in itself, shorten a person's life.

Is retinitis pigmentosa a disability?

Yes, retinitis pigmentosa (RP) can be considered a disability. Here's a breakdown:

- **Progressive Vision Loss:**
  - RP is a genetic disorder that causes progressive degeneration of the retina, leading to significant vision impairment and, in some cases, blindness.
  - This progressive nature means that the impact on an individual's vision worsens over time.
- **Impact on Daily Life:**
  - The vision loss associated with RP can severely affect a person's ability to perform everyday tasks, including:
    - Driving
    - Working
    - Navigating in low-light conditions
    - Recognizing faces
- **Legal Considerations:**
  - In many countries, including the United States, RP can qualify an individual for disability benefits under laws like the Americans with Disabilities Act (ADA).
  - The determination of disability often depends on the severity of vision loss and its impact on the individual's ability to function.
- **Social Security Disability:**
  - Individuals with RP may also be eligible for Social Security Disability Insurance (SSDI) or Supplemental Security Income (SSI) if their vision loss prevents them from working.

In essence, because retinitis pigmentosa can substantially limit major life activities, it is considered a disability.

What does a person with retinitis pigmentosa see?

Retinitis pigmentosa (RP) affects the retina's light-sensitive cells, leading to progressive vision loss. Because it affects different cells in the retina, the visual changes vary, but some common visual problems that people with RP experience are:

- **Night blindness:**
  - This is often one of the earliest symptoms. Difficulty seeing in dim light or at night is a hallmark of RP.
- **Loss of peripheral vision (tunnel vision):**
  - As the disease progresses, the field of vision narrows, resulting in "tunnel vision," where only the central vision remains. This makes it difficult to see things to the sides, above, or below.

- **Problems with central vision:**
  - In later stages, central vision can also be affected, making tasks like reading or recognizing faces challenging.
- **Color vision problems:**
  - Some individuals with RP may experience difficulty distinguishing certain colors.

In essence, a person with RP experiences a gradual loss of vision, starting with night vision and peripheral vision, and potentially progressing to central vision loss.

How quickly does retinitis pigmentosa progress?

The progression of retinitis pigmentosa (RP) varies significantly from person to person. Here's a breakdown of what influences its progression:

- **Variability:**
  - RP is a group of genetic disorders, and the rate of vision loss differs depending on the specific genetic mutation involved.
  - Therefore, there's no single timeline for how quickly RP progresses.
  - Some individuals experience slow, gradual vision loss over many years, while others may have a more rapid decline.
- **Typical Progression:**
  - Generally, RP begins with night blindness (difficulty seeing in dim light).
  - This is followed by a gradual loss of peripheral vision, leading to "tunnel vision."
  - Eventually, central vision may also be affected.
- **Factors Affecting Progression:**
  - **Genetic factors:** The specific gene mutation plays a crucial role.
  - **Individual variability:** Even with the same genetic mutation, progression can vary.
- **Key Points:**
  - Because of the variability, it is very important that people with RP have regular examinations by an ophthalmologist.
  - Monitoring the progression of RP can best be achieved by combining assessments of both visual function and morphology.

In summary, the speed of RP progression is highly individual.

What if a person suffering from retinitis pigmentosa develops cataract?

When a person with retinitis pigmentosa (RP) develops a cataract, it presents a complex situation that requires careful consideration. Here's a breakdown of the key aspects:

**Understanding the Combination:**

- **Retinitis Pigmentosa (RP):**
  - This is a progressive, degenerative disease that affects the retina, leading to a gradual loss of vision, particularly night vision and peripheral vision.
- **Cataract:**
  - This is the clouding of the eye's lens, which can further diminish vision.

**Challenges and Considerations:**

- **Increased Visual Impairment:**
  - The combination of RP and cataracts can significantly worsen visual impairment, as both conditions affect clarity of vision.
- **Surgical Considerations:**
  - Cataract surgery can be performed on individuals with RP, but it requires careful preoperative evaluation and meticulous surgical technique.
  - Patients with RP may have increased risks of certain complications during and after cataract surgery, such as:
    - Zonular weakness (weakness of the fibers that hold the lens in place).
    - Cystoid macular edema (swelling in the macula).
    - Posterior capsular opacification.
- **Expected Outcomes:**
  - While cataract surgery can improve central vision in individuals with RP, it's important to understand that it will not reverse the vision loss caused by RP itself (night vision and peripheral vision loss).
  - The degree of visual improvement after cataract surgery is variable, and is dependant on the extant damage caused by the RP.
- **Preoperative evaluation:**
  - Integrity of the ellipsoid zone and external limiting membrane on preoperative optical coherence tomographic examination are the main predictors of visual outcomes following cataract surgery.

**Key Points:**

- Cataract surgery can be beneficial for individuals with RP, but careful assessment and management are crucial.
- The focus of the surgery is to improve central vision, which is often affected by cataracts.
- It is very important for patients with RP and cataracts to have their eye care managed by an ophthalmologist that has experience with these combined conditions.

I hope this information is helpful.

Does "night-blindness" mean a person has retinitis pigmentosa?

It's important to understand that while night blindness is a common symptom of retinitis pigmentosa (RP), it doesn't automatically mean a person has RP. Here's a breakdown:

- **Retinitis Pigmentosa (RP):**
  - RP is a group of genetic disorders that affect the retina.
  - A hallmark symptom of RP is night blindness, especially in the early stages.
  - RP also leads to progressive loss of peripheral vision, often resulting in "tunnel vision."
- **Night Blindness:**
  - Night blindness (nyctalopia) is difficulty seeing in low-light conditions.
  - While it's a key symptom of RP, it can also be caused by other factors, including:
    - Vitamin A deficiency.
    - Other eye conditions, such as cataracts or glaucoma.
    - Certain medications.

In summary:

- RP commonly causes night blindness.
- However, night blindness can have other causes.
- Therefore, if someone is experiencing night blindness, it is very important that they have their eyes examined by an eye care professional, in order to get a proper diagnosis.

I hope this helps.

Which doctor should you consult for retinitis pigmentosa?

When dealing with retinitis pigmentosa (RP), it's crucial to consult with an eye specialist. Specifically:

- **Ophthalmologist:**
  - An ophthalmologist is a medical doctor specializing in eye and vision care. They are trained to diagnose and treat all eye diseases, including RP.
  - Within ophthalmology, it's particularly important to seek out a:
- **Retinal Specialist:**
  - These ophthalmologists have specialized training in diseases of the retina, which is the part of the eye affected by RP. They have the expertise to perform the necessary diagnostic tests and to provide the most current information on management and potential treatments.

Here's a breakdown of why these specialists are essential:

- **Diagnosis:**
  - They can perform the necessary examinations and tests, such as electroretinography (ERG) and visual field testing, to confirm the diagnosis of RP.
- **Management:**
  - They can help manage the symptoms and monitor the progression of the disease.
- **Information:**
  - They can provide information about current research and potential future treatments.
- **Genetic conseling:**
  - Since RP is a genetic disorder, they can give advise on genetic conseling.

Therefore, your primary point of contact should be an ophthalmologist, and ideally, one who specializes in retinal diseases.
