# Supplementary material for: Evaluation of the artificial intelligence chatbots in frequently asked questions about retinitis pigmentosa: a comparative analysis between ChatGPT-4 and Gemini-2.0
Source: Int J Retina Vitreous. 2025 Nov 28;12:1. doi: 10.1186/s40942-025-00772-4 (PMC12763912; doi:10.1186/s40942-025-00772-4)
Supplement: Supplementary file 4 — Supplementary Material 4 [file 40942_2025_772_MOESM4_ESM.docx]

What is Retinitis Pigmentosa?

Okay, imagine the back of your eye is like a movie screen, and it helps you see. Retinitis pigmentosa, or RP, is like when that movie screen starts to break down. Here's a simpler explanation:

- **What it is:**
  - RP is a group of eye problems that are passed down in families.
  - It hurts the retina, which is the part of your eye that sees light.
- **What happens:**
  - People with RP usually have trouble seeing at night first.
  - Then, they start to lose their side vision, like looking through a tunnel.
  - Over time, it can make it harder to see clearly.
- **Why it happens:**
  - It's because of problems with their genes, which they get from their parents.
- **Important things to know:**
  - There's no cure right now.
  - Doctors can help people manage it, and there are tools to help them see better.
  - It is very important to have regular eye exams, and to speak with your doctor about any family history of the disease.

Basically, RP slowly damages the part of your eye that lets you see, especially at night and in your side vision.

What causes you to get Retinitis Pigmentosa?

Okay, imagine your eyes have tiny little helpers called "retinal cells" that help you see in the dark and at the edges of your vision. Retinitis pigmentosa, or RP, happens when those helpers start to break down.

Here's the main reason why:

- **It's in your genes:**
  - Genes are like instructions passed down from your parents. Sometimes, those instructions have a typo, or a mistake.
  - These mistakes can tell your retinal cells to not work right.
  - So, if your parents had that "typo" in their genes, they might pass it on to you.
- **Different ways to get it:**
  - Sometimes, only one parent needs to pass on the "typo."
  - Other times, you need the "typo" from both parents.
  - Also sometimes the "typo" just happens, and isnt passed down from either parent.
- **What happens:**
  - Because of the "typo" in the genes, the retinal cells slowly stop working.
  - This makes it harder to see at night and in your side vision.

So, basically, RP is like a genetic mix-up that messes with the little helpers in your eyes.

Can Retinitis Pigmentosa be passed down through heredity?

Okay, so Retinitis Pigmentosa, or RP, is something you can get from your parents. It's like getting your eye color, but instead of color, it's about how your eyes work.

Here's the simple version:

- **Genes are like instructions:**
  - Your body uses genes to know how to build itself.
  - RP happens when there's a mistake in the genes that tell your eyes how to work.
- **Passed down from parents:**
  - You get half your genes from your mom and half from your dad.
  - If those genes have the RP mistake, you can get RP too.
- **Different ways to get it:**
  - Sometimes, you need the mistake from both parents.
  - Other times, you only need it from one parent.
  - Sometimes it is passed down through the X chromosome, which makes it more likely to effect boys.

Basically, RP runs in families because it's caused by those little instructions, the genes, that you get from your parents.

Can Retinitis Pigmentosa be caused by injury or accident?

Okay, here's a way to explain Retinitis Pigmentosa (RP) that's easy to understand:

Imagine your eyes have tiny little cameras inside them, called photoreceptors, that help you see. RP is a disease where these cameras start to break down.

- **Mostly from Family:**
  - Most of the time, people get RP because it runs in their family. It's like inheriting a bad instruction manual for those little cameras.
- **Not Usually from Bumps and Bruises:**
  - It's very rare for an eye injury to cause RP. Think of it like this: if you bump your knee, you don't suddenly get a disease that makes your heart stop working. It's the same with your eyes.
  - While it is not impossible, it is very unlikely that an injury is the cause.
- **So, in short:** RP is almost always from your genes, not from hurting your eye.

What are the symptoms of Retinitis Pigmentosa?

Okay, imagine your eye is like a camera, and the back of it, called the retina, is like the film. Retinitis pigmentosa, or RP, is like having that film slowly get damaged over time.

Here's what that damage can do to your eyesight:

- **Trouble seeing at night:** It's like your night vision gets turned way down. You might have a hard time seeing in movie theaters, or when you're walking around outside after dark.
- **Losing your side vision:** Imagine looking through a straw. You can see what's right in front of you, but not what's on the sides. That's kind of like how RP can make your side vision shrink, which is called "tunnel vision."
- **Blurry central vision:** Later on, it can also make the center of your vision blurry, so it's hard to read or see details.
- **Color problems:** Some people might also have trouble telling colors apart.
- **Bright light bothers you:** Some people become more sensitive to bright lights.

Basically, RP makes it harder and harder to see over time. It's important to see an eye doctor if you start having these problems.

How can Retinitis Pigmentosa be diagnosed?

Okay, imagine your eye has a camera inside called the retina. Retinitis Pigmentosa (RP) messes up that camera. So, to find out if you have RP, doctors do a few things:

- **Look inside your eye:** They use a special light to see the back of your eye, like looking through a window. They're looking for dark spots that look like tiny bones.
- **Test your eyesight:** They use eye charts to see how well you can see things far away and up close.
- **Check your side vision:** They use a machine to see how well you can see things to the side, because RP often makes it hard to see things on the edges of your vision.
- **Test your color vision:** They use special pictures with dots to see if you can tell colors apart.
- **Electrical test:** They put little stickers on your eye to see how your retina reacts to light. It's like checking the batteries in your camera.
- **Take pictures of your retina:** They use a special camera to take pictures of the layers of your retina to see if anything is damaged.
- **Check your genes:** Because RP runs in families, they might take a blood sample to see if you have the gene that causes it.
- **Ask about your family:** They will ask if anyone else in your family has vision problems.

Basically, they're checking your eye's "camera" from all angles to see if it's working right.

How does Retinitis Pigmentosa affect vision?

Okay, imagine your eye has a movie screen in the back called the retina. Retinitis pigmentosa, or RP, is like a slow-motion problem with that movie screen.

Here's how it messes with your eyesight:

- **Trouble seeing at night:**
  - Think of it like the dimmer switch on your lights getting weaker. Things get harder to see when it's dark.
- **Losing side vision:**
  - It's like looking through a tube. You can see straight ahead okay, but you can't see what's on the sides anymore. This is sometimes called "tunnel vision".
- **Blurry central vision:**
  - Later on, it can make it hard to see things right in front of you clearly, like reading or recognizing faces.
- **Colors looking weird:**
  - It can also make it harder to tell colors apart.
- **Bright lights hurt your eyes:**
  - Some people find that bright lights bother their eyes more than they used to.

So, RP slowly damages the back of your eye, making it harder and harder to see, especially at night and to your sides.

Can Retinitis Pigmentosa cause complete blindness?

Okay, imagine your eye has a movie screen in the back, called the retina. Retinitis Pigmentosa (RP) is like having that movie screen slowly start to break down.

- **Things get dark:** At first, it's like the lights in the movie theater are dimming, especially at night.
- **Tunnel vision:** Then, the edges of the movie screen start to go black, so you can only see what's right in front of you.
- **Can it go all black?:** Yes, for some people with RP, the whole movie screen can eventually go completely black. It doesn't happen to everyone, but it can.
- **Slow problem:** This breakdown happens slowly, over many years.

So, RP can make you completely blind, but it's different for everyone how fast and how much vision they lose.

Can we stop Retinitis Pigmentosa from progressing?

Okay, so retinitis pigmentosa (RP) is a condition that slowly damages your eyesight. Right now, doctors can't completely stop it, but they can do things to help it slow down and make living with it easier.

Think of it like this: Imagine your eyesight is like a sandcastle. RP is like the waves slowly washing it away. We can't stop the waves, but we can build walls to protect the sandcastle and scoop away the water as it comes in.

Here's what doctors can do:

- **Regular Checkups:** They'll keep a close eye on your vision to see how it's changing.
- **Vitamins:** Some people take special vitamins, like Vitamin A and Omega-3s, which might help slow things down a little.
- **Sunglasses:** Wearing sunglasses that block UV rays protects your eyes from more damage.
- **Tools to Help You See:** There are special glasses, magnifying tools, and other devices that can help you use the vision you still have.
- **Gene Therapy:** For some people with a specific type of RP, there's a treatment that tries to fix the problem in their genes.
- **Treating Other Eye Problems:** Sometimes, RP can cause other eye problems, like cataracts. Doctors can treat those to help your vision.

Scientists are working hard to find better treatments and even a cure. So, even though we can't stop RP completely right now, there's hope for the future. It's super important to see your eye doctor regularly if you have RP.

Can Retinitis Pigmentosa be reversed?

Okay, imagine your eyes have tiny cameras inside, and in Retinitis Pigmentosa (RP), some of those camera parts start to break down.

- **Can we fix it?** Right now, doctors can't fully fix the broken parts.
- **Can we slow it down?** Yes! There are things doctors can do to help keep the "cameras" working longer. This might include special vitamins, glasses, or learning new ways to see.
- **Is there hope?** Scientists are working hard on new ways to fix the broken parts, especially by looking at the genes (the instructions inside your body). There is even a gene therapy approved for a specific type of RP.
- **What should you do?** If you or someone you know has RP, it's important to see an eye doctor. They can help figure out the best way to take care of your eyes.

Basically, we can't rewind the damage, but we can hit the "pause" button and try to make things better.

Can sunlight exposure worsen vision of people with Retinitis Pigmentosa?

Okay, here's a simpler explanation:

Imagine your eye like a camera. Retinitis Pigmentosa (RP) slowly damages the parts of your eye that help you see, kind of like scratching the camera lens.

Now, think of sunlight like a really bright light bulb. For most people, a bright light bulb is okay. But for someone with RP, it's like shining that light directly into a scratched camera lens – it makes the picture even worse.

Here's why:

- **Eyes are extra sensitive:** RP makes eyes more sensitive to light, so even normal sunlight can feel too bright and hurt.
- **Sun can cause more damage:** The sun's rays can hurt the already damaged parts of the eye, making the RP get worse faster.
- **Glare makes it hard to see:** Bright light can cause glare, which is like trying to see through a foggy window.

So, people with RP need to protect their eyes from sunlight by wearing sunglasses and hats, just like how you'd protect a camera lens from scratches.

Can I treat Retinitis Pigmentosa by nutritional care?

Okay, so Retinitis Pigmentosa (RP) is a disease that affects your eyesight. You can't make it go away with just food, but eating the right things can help your eyes stay as healthy as possible.

Here's the simple breakdown:

- **Think of food as support, not a cure:** Eating healthy won't fix RP, but it's like giving your eyes good tools to work with.
- **Vitamin A is tricky:** Some people think Vitamin A helps, but too much can be bad. So, don't take extra Vitamin A pills unless your doctor says it's okay.
- **Omega-3s are good:** These are in fish oil and might help your eyes.
- **Eat your greens!:** Leafy greens have stuff called lutein and zeaxanthin, which are like little protectors for your eyes.
- **Eat a mix of healthy foods:** Fruits, veggies, and whole grains give your whole body, including your eyes, what they need.
- **Vitamin E can be Bad:** Some studies say that Vitamin E can actually make Retinitis Pigmentosa get worse. So avoid taking large doses of Vitamin E.
- **Talk to your eye doctor:** They know best what you should eat and if you need any extra vitamins.

Basically, eat a healthy diet, and always ask your doctor before taking any extra vitamins or making big changes to what you eat.

Is Retinitis Pigmentosa treatable?

Okay, so retinitis pigmentosa (RP) is a disease that messes with your eyesight. Sadly, doctors can't fix it completely right now. Think of it like a slow fade on a light bulb—your vision gets dimmer over time.

Here's the deal:

- **No Cure, But Help:** There's no magic pill to make RP go away. But, there are things that can help slow it down and make living with it easier.
- **Special Vitamins:** Some doctors suggest taking certain vitamins, like vitamin A, but you *have* to talk to your eye doctor first.
- **Gene Stuff:** There's one special medicine that can help people with a very rare type of RP. It works by fixing a broken gene.
- **Tools to See Better:** There are special glasses and tools that can help people with RP see better, even if their vision isn't perfect.
- **Doctors are Working On It:** Scientists are working hard to find new ways to treat RP, like fixing the broken genes or even making fake retinas.

Basically, while we can't make RP disappear yet, there are things that can help, and smart people are working on even better solutions. If you or someone you know has RP, it's super important to see an eye doctor who knows a lot about it.

What should I eat if I have Retinitis Pigmentosa?

Okay, so you've got Retinitis Pigmentosa (RP), and you want to know what to eat to help your eyes. That's smart! While food can't cure RP, it can definitely help keep your eyes as healthy as possible.

Think of it like this: your eyes need good fuel to work their best. Here's what kind of fuel they like:

- **Colorful Veggies and Fruits:**
  - Think of things like spinach, carrots, berries, and oranges. These are full of stuff called "antioxidants." Antioxidants are like tiny bodyguards that protect your eyes from damage.
- **Fatty Fish:**
  - Salmon, tuna, and mackerel are great! They have "omega-3s," which are healthy fats that help your eyes work right.
- **Vitamin A:**
  - Milk, eggs, and cheese all have Vitamin A. This vitamin is super important for seeing at night.
- **Zinc:**
  - Nuts, seeds, and whole-grain bread have zinc. Zinc helps get Vitamin A to your eyes.

**What to remember:**

- **Talk to your eye doctor:** They know your eyes best and can tell you exactly what you need.
- **Eat a mix of healthy foods:** Don't just eat one thing! Try to eat lots of different fruits, veggies, and healthy stuff.
- **Don't go overboard on sugar:** Too much sugar isn't good for anyone, including your eyes.

Basically, eat a healthy, colorful diet, and talk to your doctor. They can give you the best advice for *your* eyes.

What foods should I avoid if I have Retinitis Pigmentosa?

Okay, here's a simpler way to think about foods and Retinitis Pigmentosa (RP):

Imagine your eyes need good, clean fuel to work their best. Some foods are like dirty fuel, and they can make things harder for your eyes.

**Foods to eat less of:**

- **Fatty foods:** Like lots of cheese, hot dogs, fried chicken, and greasy chips. These can clog up your blood flow, and your eyes need good blood flow to stay healthy.
- **Sugary drinks:** Like soda and super-sweet juices. Too much sugar isn't good for your body or your eyes.
- **White stuff:** Like white bread and white rice. These foods don't have many good things in them, and they can mess with your blood sugar.

**Why?**

- RP affects your eyes, and keeping your whole body healthy helps your eyes too.
- Eating less of the bad stuff helps your blood flow better, and that's good for your eyes.
- Eating less of the bad stuff helps you keep a healthy weight, and that is good for overall health.

**What to do:**

- Talk to your eye doctor. They know the most about your eyes.
- Eating lots of fruits and vegetables is a good idea for everyone.
- Try to eat whole grain foods instead of white foods.

Basically, eat healthy, just like your doctor always says!

What is the prognosis for retinitis pigmentosa?

Okay, here's a simplified explanation of the prognosis for retinitis pigmentosa (RP):

- **What RP Does:**
  - RP is a group of eye problems that you get from your parents.
  - It slowly hurts the back of your eye, which is like the film in a camera.
  - This makes it hard to see, especially at night and on your sides.
- **How Vision Changes:**
  - At first, you might just have trouble seeing in the dark.
  - Then, your side vision starts to get worse.
  - Over time, you might get "tunnel vision," meaning you can only see straight ahead.
  - Eventually, your straight-ahead vision can also get bad.
  - It is rare for people with RP to become completely blind.
- **What Doctors Can Do:**
  - Right now, there's no cure for RP.
  - But, doctors can help you use special tools to see better.
  - Also, there are some treatments that can slow down how fast your vision gets worse.
  - There is also gene therapy available for some people with a specific genetic cause of RP.
  - It is very important to see an eye doctor on a regular basis.
- **Important Points:**
  - How fast your vision gets worse is different for everyone.
  - Even though RP can cause serious vision problems, many people keep some of their eyesight for a long time.
  - There is on going research being done to find new treatments.

In simple terms, RP causes vision to slowly get worse, but there are ways to help, and scientists are working on better treatments.

Can people with retinitis pigmentosa drive?

Retinitis pigmentosa (RP) messes with your eyesight, and it gets worse over time. Imagine your eyes are like a camera, and RP slowly breaks the parts that let you see clearly, especially at night and to the sides.

**Can you drive with it?**

- **Early on:** Maybe. Some people with RP can still drive, especially during the day.
- **Later on:** Probably not. Because RP makes it hard to see at night and to see things coming from the sides, driving becomes very dangerous.
- **Think about this:**
  - It's like trying to drive with blinders on, and only being able to see what's right in front of you.
  - Night driving becomes super risky.
  - Even daytime driving can get tough.

**What should you do?**

- Get your eyes checked a lot.
- Follow your state's driving rules.
- If your doctor says it's not safe, don't drive.
- There are other ways to get around, like buses, trains, or asking friends and family for rides.

Basically, RP and driving don't mix well in the long run. It's all about staying safe.

Does pregnancy have an effect on retinitis pigmentosa?

Okay, so, retinitis pigmentosa (RP) is an eye disease that messes with your vision. If you're a woman with RP and you get pregnant, things can get a little tricky.

Basically, being pregnant *might* make your RP worse. Some studies show that after having babies, some women with RP have a harder time seeing clearly, and their side vision might shrink. It's like your eyes get a little weaker.

Scientists aren't 100% sure why this happens. They think it might have something to do with all the hormone changes that happen when you're pregnant. It's like those hormones can sometimes stir up the RP and make it progress faster.

So, if you have RP and you're thinking about having a baby, it's super important to talk to your eye doctor and your baby doctor. They can help you understand the risks and keep an eye on your vision during your pregnancy. They can also help make a plan to keep your vision as good as possible.

What is the likelihood that I will pass the retinitis pigmentosa gene on to my children?

Okay, let's break down how you might pass retinitis pigmentosa (RP) to your kids, in simple terms:

Imagine your genes are like instructions in a recipe book. Sometimes, there's a typo in the recipe, and that typo can cause RP.

- **If it's a "strong" typo (dominant):**
  - If you have this strong typo, it's like having a recipe where even one small error ruins the whole dish.
  - Each of your kids has a 50/50 chance of getting that typo from you.
- **If it's a "weak" typo (recessive):**
  - This is like having a recipe where you need *two* typos to mess things up.
  - If you have two weak typos, you have RP.
  - If your partner doesn't have any typos, your kids will just carry one typo, but probably won't have RP.
  - If your partner also has one of these typos, then there is a 25% chance the kid gets two typos and has RP, a 50% chance the kid gets one typo and is a carrier, and a 25% chance the kid gets no typos.
- **If it's on the "X" gene:**
  - This is a bit more complicated, because boys and girls get different "X" genes.
  - If mom has the typo, sons have a 50% chance of getting RP, and daughters have a 50% chance of carrying the typo.
  - If dad has the typo, all daughters will carry the typo, and sons will not get it.
- **Sometimes, it just happens:**
  - Like a random typo appearing in a new cookbook, RP can happen even if no one else in your family has it.

**The most important thing to remember:**

- Get a doctor to do a gene test. This will tell you exactly what kind of "typo" you have.
- Talk to a genetic counselor. They can explain your risks clearly.

Think of the genetic counselor as a translator, they can translate all the complicated doctor talk into easy to understand information.

What should I do if my child is diagnosed with retinitis pigmentosa?

Okay, imagine your child's eyes are like a camera, and retinitis pigmentosa (RP) is like a problem with the film in that camera. It makes it harder for the camera to take good pictures, especially in dim light.

Here's what you should do:

**1. Find a Special Eye Doctor:**

- You need to see an eye doctor who knows a lot about RP. They can tell you exactly what's going on and how to help.
- Also, talk to a "genetic counselor." They can explain why this happened and if other family members might have it too.

**2. Learn About RP:**

- RP makes it hard to see at night and in your side vision.
- You might also have trouble seeing colors or be bothered by bright lights.
- Websites like the Foundation Fighting Blindness are great for learning more.

**3. Help Your Child:**

- If your child's vision gets worse, get help from people who know how to teach kids with low vision.
- Talk to your child's school. They can help with things like special seating or tools that make it easier to learn.
- It's okay for your child to feel sad or scared. Talk to a counselor or join a support group.
- Help your child do things on their own, even if it's a little harder.

**4. Keep Up With New Treatments:**

- Scientists are working hard to find ways to help people with RP.
- There are special tools that can help your child see better.
- Eating healthy and staying active is always a good idea.

**Important things to remember:**

- Getting your child's genes tested can be very helpful.
- Organizations like the foundation fighting blindness are a great resource.
- Keep in close contact with your child's eye doctor.

Think of it like this: even if the camera's film isn't perfect, there are ways to take good pictures and enjoy life.

What is the extent of independence for people with advanced retinitis pigmentosa?

Okay, let's break down how retinitis pigmentosa (RP) affects someone's ability to live independently, in a simple way:

Imagine your eyesight is like a camera. RP is like that camera slowly breaking down. Here's what that means:

- **What RP does:**
  - It's a disease that damages the back of your eye, where the cells that help you see are.
  - This causes your vision to get worse over time.
  - First, you might have trouble seeing at night.
  - Then, you might lose your side vision, like looking through a tunnel.
  - Eventually, it can make it hard to see things right in front of you.
- **How it affects daily life:**
  - Things like driving, reading, and moving around can become very difficult.
  - It can be hard to do everyday tasks, like cooking or shopping.
- **But, people can still live independently:**
  - There are tools that help:
    - Magnifying glasses and special devices can make things look bigger.
    - Technology like screen readers can help people use computers.
    - Training can help people learn to use canes or work with guide dogs to get around safely.
  - There are also people who can help:
    - Therapists can teach ways to do things differently.
    - Support groups let people share tips and feelings.
- **Basically:**
  - RP makes seeing hard, but it doesn't mean people can't live their lives.
  - With the right tools and help, they can still do a lot of things on their own.

So, while RP creates challenges, there's a lot that can be done to help people stay independent.

What part of the eye is affected by retinitis pigmentosa?

Okay, here's a simple explanation:

- **The Retina:**
  - Think of the retina as the "film" at the back of your eye. It's the part that captures light, like how a camera captures a picture.
  - The retina has special cells, kind of like tiny light sensors, called "rods" and "cones."
- **What Retinitis Pigmentosa Does:**
  - Retinitis pigmentosa (RP) is a disease that damages those "rod" and "cone" cells in the retina.
  - It mostly hurts the "rod" cells first, which are important for seeing at night and seeing things to the side.
  - Later on, it can also hurt the "cone" cells, which help you see clearly straight ahead and see colors.
  - In short, RP damages the part of your eye that sees light, causing vision to deteriorate.

Can women get retinitis pigmentosa?

Okay, let's break down retinitis pigmentosa (RP) in a simple way:

- **What is RP?**
  - RP is an eye problem that's passed down in families. It messes with the retina, which is the part of your eye that helps you see.
  - It slowly makes it harder to see, especially at night and in your side vision.
- **Can women get it?**
  - Yes, women can get RP.
  - Sometimes, how it shows up in women is different than in men, because of how they inherit it.
  - Because women have two X chromosomes, they can be a "carrier" of the gene that causes RP. This means that they might have the gene, and be able to pass it on to their children, but may not have very severe symptoms themselves.
  - But in some cases, women do have very serious vision loss from RP.
- **Important to know:**
  - If RP runs in your family, it's a good idea to talk to a doctor.
  - Genetic counseling can help people understand the risks of passing the condition on to their children.

How long is the average lifespan of a person with retinitis pigmentosa?

Okay, here's a simpler way to understand it:

- **Retinitis pigmentosa (RP) is an eye problem.** It mainly affects how well someone can see.
- **Having RP usually doesn't make someone live a shorter life.** Most people with RP live just as long as anyone else.
- **Think of it this way:** RP affects the eyes, not the whole body. So, while it changes how someone sees, it doesn't usually change how long they live.
- It is important to remember that in rare cases, RP is connected to other health problems, and those other health problems could have an impact on a persons health.

So, in short, if someone has RP, they can expect to live a normal lifespan.

Is retinitis pigmentosa a disability?

Okay, so retinitis pigmentosa (RP) is a disease that messes with your eyes. Imagine the back of your eye like a movie screen; RP slowly damages that screen.

Here's why it's a disability:

- **Your eyesight gets worse over time.** It's not like you just wake up one day and can't see. It's a slow process that makes it harder and harder to see.
- **It makes everyday stuff really hard.** Things like driving, reading, or even just walking around at night become super difficult.
- **The government sees it as a disability.** If your eyesight gets bad enough, you might qualify for help from the government, like getting money to help you live.

Basically, RP can make it so you can't do things most people can do, and that's why it's considered a disability.

What does a person with retinitis pigmentosa see?

Imagine your eye is like a camera, and the back of your eye, the retina, is like the film. Retinitis pigmentosa, or RP, is like the film slowly getting damaged.

Here's how that damage affects what you see:

- **Trouble seeing at night:** It's like trying to take a picture in a dark room without a flash. Everything looks very dark and blurry.
- **Losing your side vision:** Imagine looking through a tube. You can see what's right in front of you, but you can't see anything to the sides. This is called "tunnel vision."
- **Blurry center vision:** Later on, even what's straight ahead can get blurry, making it hard to read or see faces clearly.
- **Colors looking off:** Some people with RP also have trouble telling certain colors apart.

So, RP slowly makes it harder to see, like the "film" in your eye is fading away bit by bit.

How quickly does retinitis pigmentosa progress?

Imagine your eyes have a camera inside them, and retinitis pigmentosa (RP) is like dust slowly covering the lens. How fast the dust builds up is different for everyone.

- **Some people:** The dust builds up very slowly, over many, many years. They might notice it's harder to see at night first.
- **Other people:** The dust builds up faster, and their vision gets worse more quickly.
- **What happens:**
  - First, it gets hard to see in the dark.
  - Then, you start losing your side vision, like looking through a tunnel.
  - Finally, some people lose their sharp, center vision too.

So, how fast it happens depends on the person. There's no one answer. It's really important to see an eye doctor regularly if you have RP, so they can keep an eye on how things are changing.

What if a person suffering from retinitis pigmentosa develops cataract?

Okay, imagine your eye like a camera. The lens in your eye helps you focus, just like a camera lens.

- **Retinitis Pigmentosa (RP):** This is like a problem with the film in your camera. The film gets damaged over time, making it hard to see, especially at night and to the sides.
- **Cataracts:** This is like your camera lens getting cloudy. It makes everything look blurry.

So, if you have RP (damaged film) and get cataracts (cloudy lens), your vision gets even worse.

**What happens next?**

- Doctors can do surgery to fix the cloudy lens (cataract surgery). This can make your central vision clearer.
- But, the surgery won't fix the damaged film (RP). So, you'll still have trouble seeing at night and to the sides.
- It's important to see an eye doctor who knows how to deal with both problems. They'll check your eyes carefully before surgery and help you understand what to expect.

Basically, cataract surgery can help with the blurriness, but it won't cure the underlying RP.

Does "night-blindness" mean a person has retinitis pigmentosa?

Okay, so "night blindness" means you have trouble seeing when it's dark. Think of it like trying to find your way around your house at night without any lights on – it's really hard!

Now, a disease called retinitis pigmentosa (RP) often causes night blindness. RP is like a problem with the back of your eye, where the part that helps you see is located.

But, just because you have trouble seeing at night doesn't automatically mean you have RP. There are other things that can cause night blindness too, like:

- Not getting enough vitamin A (like from carrots!).
- Having other eye problems, like cataracts (cloudy eyes).
- Taking certain medicines.

So, if you're having trouble seeing at night, it's important to go see an eye doctor. They can figure out what's going on and help you see better.

Which doctor should you consult for retinitis pigmentosa?

If you think you might have retinitis pigmentosa (RP), or if you've been told you have it, you need to see a special eye doctor. Here's who can help:

- **Eye Doctor (Ophthalmologist):**
  - This is a doctor who knows all about eyes and how they work. They can check your eyes and tell you what's going on.
- **Retina Expert (Retinal Specialist):**
  - Inside your eye, there's a part called the retina. RP messes with the retina. A retina expert is an eye doctor who knows a lot about the retina and diseases that affect it. They can help you understand RP and what you can do.

Think of it this way:

- An eye doctor is like a general doctor for your eyes.
- A retina expert is like a specialist, like a heart doctor or brain doctor, but for the back of your eye.

So, start by seeing an eye doctor, and they can send you to a retina expert if needed. They can help you with tests, tell you what's happening, and give you information about RP.
